# Novel cellulose supported 1,2-bis(4-aminophenylthio)ethane Ni(ii) complex (Ni^II^(BAPTE)(NO_3_)_2_-Cell) as an efficient nanocatalyst for the synthesis of spirooxindole derivatives†

**DOI:** 10.1039/d1ra08182a

**Published:** 2022-01-27

**Authors:** Raziyeh Keshavarz, Mahnaz Farahi

**Affiliations:** Department of Chemistry, Yasouj University Yasouj Iran 75918-74831 farahimb@yu.ac.ir +987412242167e

## Abstract

Cellulose was used as a support for immobilizing a Ni(ii) complex of 1,2-bis(4-aminophenylthio)ethane to prepare Ni^II^(BAPTE)(NO_3_)_2_-Cell as a new organo–inorganic hybrid nanocatalyst. The properties of the prepared catalyst were studied using various analyses such as FT-IR, XRD, SEM, TGA and EDX. Ni^II^(BAPTE)(NO_3_)_2_-Cell was employed as a reusable catalyst for the synthesis of spirooxindole derivatives *via* a three-component condensation of isatin, malononitrile and reactive methylene compounds. The nanocatalyst can be readily and quickly separated from the reaction mixture and can be reused for at least eight successive reaction cycles without a significant reduction in efficiency. The facile accessibility to the starting materials, use of green solvents and conducting the reactions in eco-friendly and cost-effective conditions have made this protocol a suitable method for preparing spirooxindole derivatives.

## Abstract 2

Ni^II^(BAPTE)(NO_3_)_2_-Cell as a novel cellulose supported catalyst has been prepared and characterized by various techniques including FT-IR, XRD, SEM, TGA and EDX.

## Graphical abstract

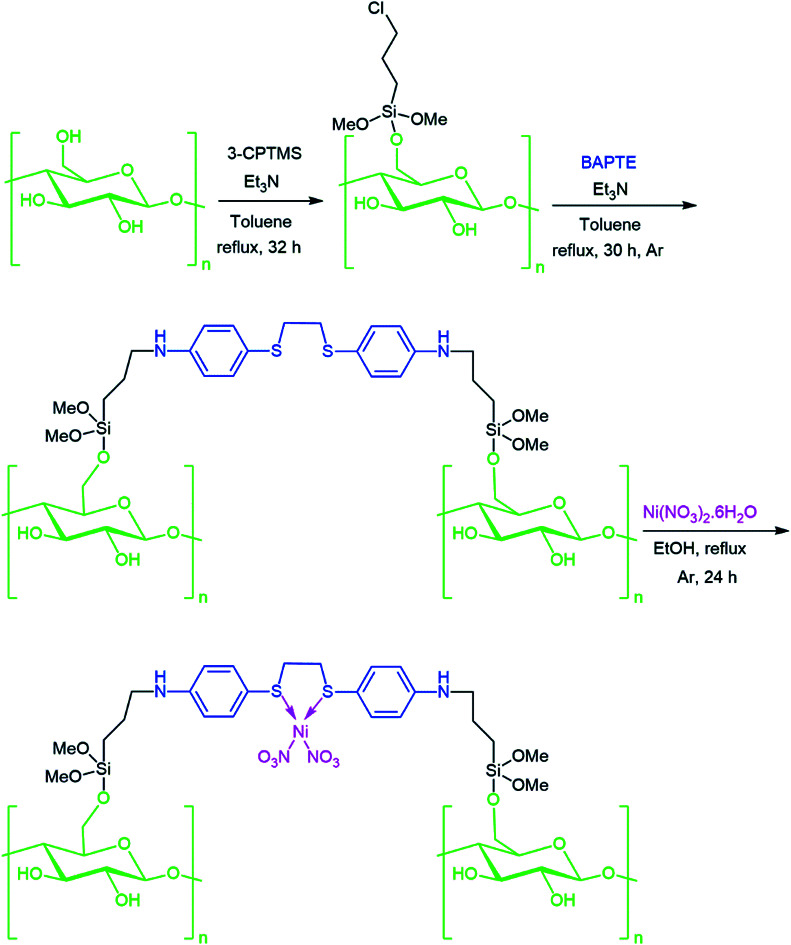

## Introduction

The development of catalytic reactions is one of the requirements of environmentally friendly processes.^1^ Many chemical reactions are not possible without the presence of a catalyst.^2^ Therefore, the use of green and recyclable catalysts has attracted a lot of attention. The three most important classes of these substances are enzymes, homogeneous catalysts and heterogeneous catalysts.^3^ Enzymes have been used as biocatalysts in biochemical and chemical processes for a long time.^4,5^ Homogeneous catalysts have better performance than heterogeneous catalysts due to their better dispersion and co-phase with the reaction mixture, but the problem with their use is that it is difficult and almost impossible to separate them from the reaction medium and reuse them.^6–10^ Immobilizing homogeneous catalysts on various supports, such as carbon,^11^ silica,^12,13^ metal oxide,^14,15^ polymer,^16–18^ and nanocomposites,^19–21^ is one of the efficient ways to overcome this problem. Carbon-based materials, due to their unique properties such as light weight, many varieties of forms, doping capability with hetero atoms, low-cost and ease of processability, are suitable supports for heterogeneous catalysts.^22–24^ Hydrocarbons such as chitosan,^25,26^ starch,^27–29^ gelatin,^30–32^ alginate^33,34^ and cellulose^35,36^ can be used as low-cost supports for the synthesis of heterogeneous catalysts. Among them, cellulose has received a lot of consideration due to its large surface area, good mechanical properties and almost inexhaustible, biodegradable, and renewable properties.^37–39^ Cellulose polysaccharide ((C_6_H_10_O_5_)_*n*_), a biomass-derived material, the most widespread reusable carbon, natural polymer and common carbon-based material, is abundant in nature and there are several ways to separate it. This polysaccharide compound has an acceptable resistance to chemical changes in reactions due to its beta-(1–4) glycosidic link. In addition, its ease of availability, cheapness, and reusability make it a green catalyst support. It is biodegradable and does not produce biowaste.^40–45^ At the nanoscale, cellulose nanocrystals, in addition to their cellulose properties, also exhibit specific properties of nanomaterials, such as dispersion and greater contact surface area.^46^ Cellulose with a modified surface has many applications.^47–49^ For example, by placing a coating of aluminum oxide on the surface of cellulose, a suitable compound is obtained to adsorb metal halides from ethanol.^50^ Also, metal nanoparticles such as Ag, Cu, Au, Pd, Pt and *etc.* loaded on cellulose nanocrystalline have been introduced and used as heterogeneous catalysts.^51–55^

Recently, inorganic/organic hybrid materials have been widely used in organic reactions as a catalyst, because they are well-matched with various processes of eco-friendly chemical transformations.^56^ This quickly growing field is producing several exciting new materials with novel properties. They gather together typical advantages of organic components like flexibility, low density, toughness, and formability, with the ones displayed with specific inorganic materials like hardness, chemical resistance, strength, optical properties, among others.^57^ The properties of these materials are not only the sum of the individual contributions of both phases, but the role of the internal interfaces could be substantial. Organic/inorganic grafted materials have appeared as a superseded material to design unique products and formed a new field of academic studies.

A major challenge for the modern chemistry is the design of efficient chemical transformation with the minimum number of synthetic steps and less time.^58–60^ Multicomponent reactions (MCRs) play a key role in combinatorial chemistry due to their advantages such as their effective atom economy, straightforward experimental procedures, time saving and convergent nature.^61–64^ These reactions represent a very useful tool for the synthesis of different fine chemicals and drug intermediates as they address essential principles of synthetic efficiency.^65,66^ These features make them suitable for the easy and effective preparation of complex heterocyclic compounds. Consequently, developing simple, novel and cost-effective multi component reactions for the preparation of new compounds is an attractive area of research in current organic chemistry.^67–70^

For many years, organic chemists made great efforts to synthesize complex chemical structures. The production of quaternary carbon compounds is one of these activities. Spiro polycyclic compounds with only one common carbon atom is an important class of organic compounds.^71^ Spirooxindole with interesting architecture, consist of two main parts, including the oxindole core and the fused part in the 3-position of rings.^72,73^ Spirooxindole scaffold is the main structural subunit in many natural products as alkaloids, terpenoids and lactones.^74–76^ These ring systems have been used regularly as a scaffold in medicinal and agricultural chemistry.^77–80^ Synthesis of spirooxindoles is an interesting subject in synthetic chemistry due to their pharmacological activities such as anti-cancer, anti-HIV, anti-tuberculosis, antifungal, antibacterial and anti-malarial properties.

In continuation of our program aimed at developing new methodologies for the preparation of green catalysts,^81–90^ we have reported preparation, characterization and catalytic application of Ni^II^(BAPTE)(NO_3_)_2_-Cell as a novel cellulose-based nanocomposite.

## Experimental

### Methods and materials

All starting chemical reagent was used without further purification and was obtained from Aldric, Fluka and Merck chemical companies. The reaction progress and purity of the compounds was monitoring by thin layer chromatography (TLC) Grade silica gel-G/UV (254 nm) plates. Melting point was taken using Electrothermal KSB1N device. JASCO FT-IR/680 instrument was applied to recorded Fourier transform infrared (FT-IR) using KBr disks. The NMR spectra were performed with a Bruker 400 Ultrashield (400 MHz/^1^H NMR and 100 MHz/^13^C NMR), with CDCl_3_ as solvent. XRD patterns were obtained using a Philips X Pert Pro X diffractometer operated with Ni-filtered Cu-Kα radiation (*λ* = 0.15418 nm) source. Thermogravimetric analysis (TGA) was conducted using a Perkin–Elmer Pyris 1 instrument at 25–900 °C. Scanning electron microscopy (SEM: KYKY-EM3200) under voltage of 26 kV was used to show the morphology and size of nanoparticles. Energy dispersive spectroscopy (EDS) was determined using the TESCAN vega model instrument.

### Synthesis of chloropropyl-Cell

Cellulose (1 g) in dry toluene (50 mL) placed on ultrasonic bath and dispersed for 15 min. Next, Et_3_N (1 mL) and 3-chloropropyl trimethoxy silane (3 mL) were added and the resulting mixture was stirred vigorously under reflux condition at Ar atmosphere for 32 h. After this time, the suspension was filtered and the product was washed three time by EtOH and dried at room temperature for 24 h.^91^

### Preparation of BAPTE-Cell

Chloropropyl-Cell (1 g) was treated by 1,2-bis(4-aminophenylthio)ethane ligand (100 mg) in the presence of trimethylamine (3 mL) in dry toluene (250 mL) and refluxed under Ar atmosphere for 30 h. Then, the generated products were collected by filtrations and washed several times with anhydrous toluene and then ethanol, and then dried.^92^

### Preparation of Ni^II^(BAPTE)(NO_3_)_2_-Cell nanocatalyst 1

A mixture of Ni(NO_3_)_2_·6H_2_O (0.25 g) and BAPTE-Cell (0.40 g) in absolute EtOH (60 mL) was refluxed under Ar atmosphere for 24 h. Next, the resulting mixture was filtered and Ni^II^(BAPTE)(NO_3_)_2_-Cell nanocatalyst was separated and washed with EtOH three time and dried at 70 °C.

### General procedure for the synthesis of spirooxindole derivatives 5 by Ni^II^(BAPTE)(NO_3_)_2_-Cell nanocatalyst 1

Nanocatalyst 1 (0.008 g) was added to a mixture of isatin 2 (1 mmol), malononitrile 3 (1 mmol) and compound 4 (1 mmol) in water (5 mL). The mixture was stirred under reflux condition. After completion of the reaction, boiling ethanol (5 mL) was added and the catalyst was separated by filtration. After cooling, the product was collected and washed with H_2_O and EtOH. Finally, the pure product was obtained by recrystallization from EtOAc.

#### 2-Amino-7,7-dimethyl-2′,5-dioxo-5,6,7,8-tetrahydrospiro[chromene-4,3′-indoline]-3-carbonitrile 5a

Mp: 290–292 °C, IR (KBr): *v*_max_ = 3376, 3313, 3145, 2960, 2192, 1722, 1681, 1656, 1604, 1471, 1348, 1222, 1054, 902 cm^−1^. ^1^HNMR (400 MHz, DMSO-*d*_6_): *δ* = 10.41 (s, 1H, NH), 7.25 (s, 2H, NH_2_), 7.13–7.17 (m, 1H, arom. CH), 6.99 (d, 1H, *J* = 7.2 Hz, arom. CH), 6.90 (t, 1H, *J* = 7.4 Hz, CH_2_), 6.80 (d, 1H, *J* = 8.0 Hz, arom. CH), 2.58 (s, 2H, CH_2_), 2.17 (s, 2H, CH_2_), 1.05 (s, 6H, CH_3_) ppm; ^13^C NMR (100 MHz, DMSO-*d*_6_): *δ* = 195.36, 178.50, 164.62, 159.24, 142.53, 134.89, 128.64, 123.50, 122.15, 117.83, 111.26, 109.71, 57.93, 50.46, 47.28, 32.43, 28.08, 27.49 ppm.

#### 2′-Amino-2,5′-dioxo-5′*H*-spiro[indoline-3,4′-pyrano[3,2-*c*]chromene]-3′-carbonitrile 5b

Mp: 285–287 °C, IR (KBr): *v*_max_ = 3363, 3274, 2202, 1720, 1670, 1612, 1207, 1106, 960 cm^−1^. ^1^HNMR (400 MHz, DMSO-*d*_6_): *δ* = 10.73 (s, 1H, NH), 7.97 (m, 2H, arom. CH), 7.78 (m, 1H, arom. CH), 7.71 (s, 2H, NH_2_), 7.54 (m, 2H, arom. CH), 7.24 (m, 2H, arom. CH), 6.93 (m, 2H, arom. CH) ppm; ^13^C NMR (100 MHz, DMSO-*d*_6_): *δ* = 177.69, 158.94, 158.79, 155.59, 152.52, 142.65, 134.18, 133.54, 129.44, 125.51, 124.61, 123.19, 122.59, 117.48, 117.15, 112.99, 110.03, 101.88, 57.49, 48.10 ppm.

#### 7′-Amino-2,2′,4′-trioxo-1′,2′,3′,4′-tetrahydrospiro[indoline-3,5′-pyrano[2,3-*d*]pyrimidine]-6′-carbonitrile 5c

Mp: 275 °C, IR (KBr): *v*_max_ = 3413, 3297, 3162, 2198, 1716, 1685, 1535, 1326, 1168, 1068, 929 cm^−1^. ^1^HNMR (400 MHz, DMSO-*d*_6_): *δ* = 12.31 (s, 1H, NH), 11.16 (s, 1H, NH), 10.52 (s, 1H, NH), 7.40 (s, 2H, NH_2_), 7.17 (m, 2H, arom. CH), 6.93 (t, 1H, *J* = 7.0, arom. CH), 6.81 (m, 1H, arom. CH), ppm; ^13^C NMR (100 MHz, DMSO-*d*_6_): *δ* = 178.15, 161.90, 158.72, 153.81, 149.71, 142.55, 133.95, 128.99, 124.23, 122.29, 117.41, 109.77, 87.26, 58.23, 47.11 ppm.

#### 2-Amino-2′,5-dioxo-5,6,7,8-tetrahydrospiro[chromene-4,3′-indoline]-3-carbonitrile 5d

Mp: >300 °C, IR (KBr): *v*_max_ = 3347, 3297, 3174, 2950, 2198, 1708, 1681, 1612, 1465, 1315, 1141, 1076, 937 cm^−1^. ^1^HNMR (400 MHz, DMSO-*d*_6_): *δ* = 10.42 (s, 1H, NH), 7.25 (s, 2H, NH_2_), 7.16 (m, 1H, arom. CH), 7.02 (dd, 1H, arom. CH), 6.91 (m, 1H, arom. CH), 6.80 (d, 1H, *J* = 7.0, arom. CH), 2.68 (m, 2H, CH_2_), 2.24 (m, 2H, CH_2_), 1.94 (m, 2H, CH_2_) ppm; ^13^C NMR (100 MHz, DMSO-*d*_6_): *δ* = 195.49, 178.60, 166.51, 159.10, 142.47, 135.02, 128.61, 123.67, 122.10, 117.85, 112.35, 109.61, 57.99, 47.35, 36.85, 27.21, 20.27 ppm.

#### 7′-Amino-1′,3′-dimethyl-2,2′,4′-trioxo-1′,2′,3′,4′-tetrahydro spiro[indoline-3,5′-pyrano[2,3-*d*]pyrimidine]-6′-carbonitrile 5e

Mp: 230–231 °C, IR (KBr): *v*_max_ = 3385, 3240, 2960, 2202, 1702, 1640, 1496, 1380, 1046, 931 cm^−1^. ^1^HNMR (400 MHz, DMSO-*d*_6_): *δ* = 10.53 (s, 1H, NH), 7.59 (s, 2H, NH_2_), 6.81–7.21 (m, 4H, arom. CH), 3.04 (s, 3H, CH_3_), 3.19 (s, 3H, CH_3_) ppm; ^13^C NMR (100 MHz, DMSO-*d*_6_): *δ* = 178.06, 159.90, 158.55, 152.50, 150.13, 142.58, 134.06, 128.95, 124.26, 122.25, 117.29, 109.75, 87.53, 58.16, 56.52, 29.83, 28.09 ppm.

#### Ethyl 2′-amino-3′-cyano-6′-methyl-2-oxospiro[indoline-3,4′-pyran]-5′-carboxylate 5f

Mp: 263–265 °C, IR (KBr): *v*_max_ = 3390, 3259, 3142, 2954, 2206, 1724, 1677, 1619, 1465, 1334, 1149, 1066, 968 cm^−1^. ^1^HNMR (400 MHz, DMSO-*d*_6_): *δ* = 10.43 (s, 1H, NH), 7.31 (s, 2H, NH_2_), 6.80–7.21 (m, 4H, arom. CH), 3.50 (q, 2H, CH_2_), 2.10 (s, 3H, CH_3_), 1.08 (t, 3H, CH_3_) ppm; ^13^C NMR (100 MHz, DMSO-*d*_6_): *δ* = 178.98, 165.58, 159.41, 158.93, 142.42, 134.90, 129.05, 123.83, 122.35, 117.95, 109.78, 105.34, 56.94, 51.85, 49.52, 19.33, 19.29 ppm.

#### Methyl 2′-amino-3′-cyano-6′-methyl-2-oxospiro[indoline-3,4′-pyran]-5′-carboxylate 5g

Mp: 279–281 °C, IR (KBr): *v*_max_ = 3390, 3239, 2954, 2202, 1720, 1673, 1623, 1469, 1330, 1145, 1076, 975 cm^−1^. ^1^HNMR (400 MHz, DMSO-*d*_6_): *δ* = 10.43 (s, 1H, NH), 7.22 (s, 2H, NH_2_), 6.80–7.20 (m, 4H, arom. CH), 3.52 (s, 3H, CH_3_), 2.11 (s, 3H, CH_3_) ppm; ^13^C NMR (100 MHz, DMSO-*d*_6_): *δ* = 178.97, 165.58, 159.41, 158.92, 142.42, 134.90, 129.05, 123.84, 123.15, 122.35, 109.78, 105.34, 54.94, 51.90, 49.52, 19.20 ppm.

#### 2-Amino-2′,5-dioxo-6,7-dihydro-5*H*-spiro[cyclopenta[*b*]pyran-4,3′-indoline]-3-carbonitrile 5h

Mp: >300 °C, IR (KBr): *v*_max_ = 3342, 3229, 3122, 2955, 2192, 1717, 1663, 1895, 1465, 1334, 1118, 1035 cm^−1^. ^1^HNMR (400 MHz, DMSO-*d*_6_): *δ* = 10.42 (s, 1H, NH), 7.42 (s, 2H, NH_2_), 7.03–7.27 (m, 4H, arom. CH), 2.64 (m, 2H, CH_2_), 2.24 (m, 2H, CH_2_), ppm; ^13^C NMR (100 MHz, DMSO-*d*_6_): *δ* = 195.50, 178.61, 166.52, 159.10, 142.47, 135.02, 128.61, 123.67, 122.10, 117.85, 112.35, 109.62, 57.98, 47.35, 36.84, 27.21 ppm.

#### 2′-Amino-5′-benzoyl-6′-methyl-2-oxospiro[indoline-3,4′-pyran]-3′-carbonitrile 5i

Mp: 260–262 °C, IR (KBr): *v*_max_ = 3340, 3257, 3172, 2952, 2198, 1706, 1680, 1462, 1355, 1157, 1038, 927 cm^−1^. ^1^HNMR (400 MHz, DMSO-*d*_6_): *δ* = 10.40 (s, 1H, NH), 6.73–7.82 (m, 9H, arom. CH), 6.58 (s, 2H, NH_2_), 1.71 (s, 3H, CH_3_) ppm; ^13^C NMR (100 MHz, DMSO-*d*_6_): *δ* = 194.73, 183.12, 164.23, 151.10, 138.29, 134.62, 133.24, 129.36, 129.27, 128.91, 127.41, 126.30, 123.39, 119.12, 113.53, 112.11, 55.58, 54.09, 25.90 ppm.

#### 6′-Amino-3′-methyl-2-oxo-1′-phenyl-1′*H*-spiro[indoline-3,4′-pyrano[2,3-*c*]pyrazole]-5′-carbonitrile 5j

Mp: 239–240 °C, IR (KBr): *v*_max_ = 3459, 3297, 3174, 3070, 2923, 2194, 1700, 1650, 1612, 1465, 1326, 1126, 1072, 933 cm^−1^. ^1^HNMR (400 MHz, DMSO-*d*_6_): *δ* = 10.78 (s, 1H, NH), 7.61 (s, 2H, NH_2_), 6.95–7.83 (m, 9H, arom. CH), 1.57 (s, 3H, CH_3_) ppm; ^13^C NMR (100 MHz, DMSO-*d*_6_): *δ* = 177.99, 161.50, 145.40, 144.42, 142.07, 137.71, 132.60, 129.94, 129.76, 127.05, 125.38, 123.12, 120.60, 118.44, 110.32, 96.82, 56.61, 48.25, 12.17 ppm.

### Reusability of the catalyst

A mixture of Ni^II^(BAPTE)(NO_3_)_2_-Cell nanocatalyst (0.008 g), isatin (1 mmol), malononitrile (1 mmol) and dimedone (1 mmol) in H_2_O (5 mL) was refluxed for 5 min. Next, boiling ethanol (5 mL) was added to the reaction and the catalyst was separated. The separated catalyst was washed with EtOH (10 mL) and deionized water (10 mL), dried at 100 °C and reused in the subsequent runs.

## Results and discussion

**Scheme 1:**
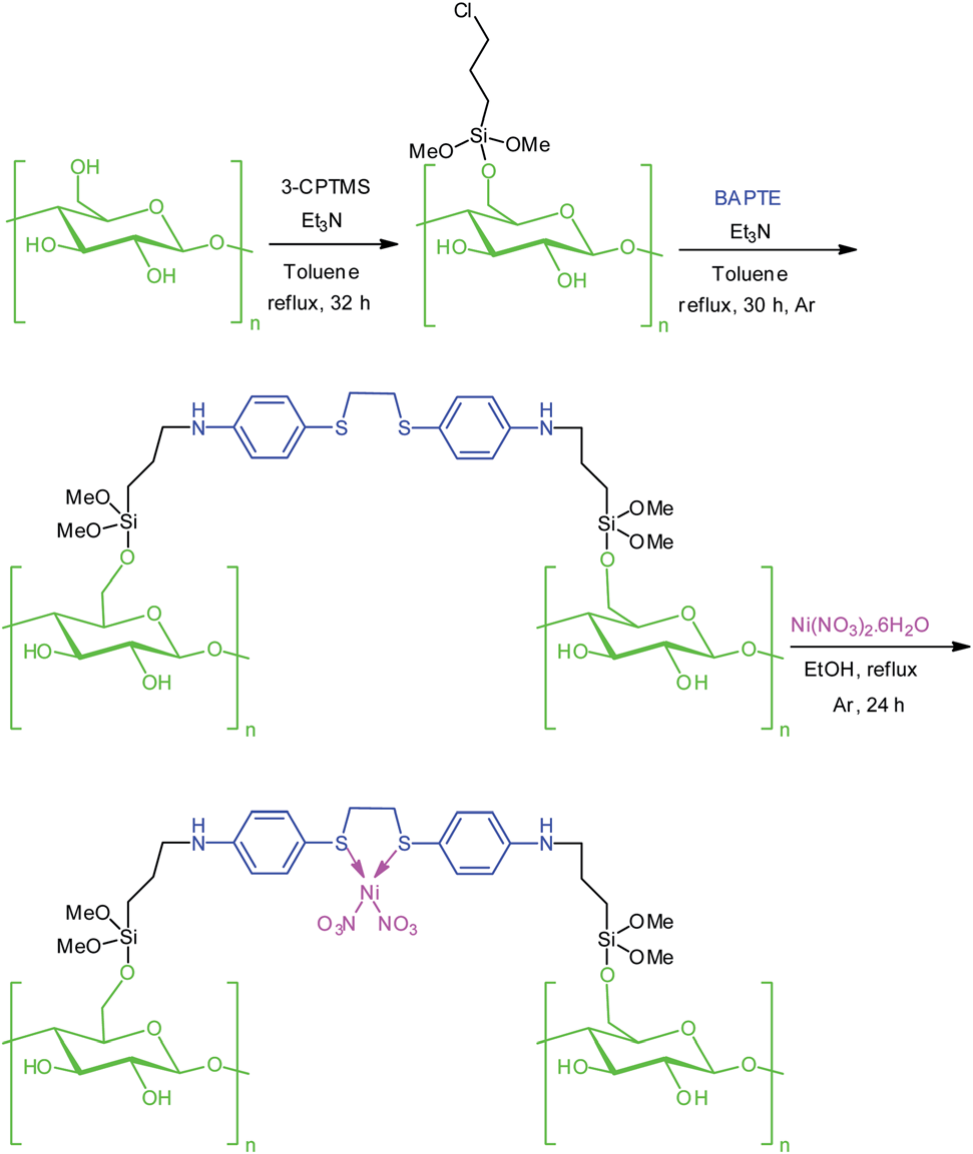
Preparation of Ni^II^(BAPTE)(NO_3_)_2_-Cell nanocatalyst 1.

In the last decade with an impressive increase in population, humanity faces concerns of environmental pollution and lack of resources and energy. So, preparation of low-cost, non-toxic and environmentally compatible catalysts have attracted significant attention. In addition, it would be economically viable if the catalyst could be reused without reducing catalytic activity. The targeted Ni^II^(BAPTE)(NO_3_)_2_-Cell as a safe and reusable nanocatalyst was prepared using a convenient procedure as illustrated in Scheme 1. Initially, the cellulose were modified with 3-chloropropyl trimethoxysilane (3-CPTMS) and then reacted with 1,2-bis(4-aminophenlythio)ethane (BAPTE) in the presence of triethylamine. Subsequently, by the addition of Ni(NO_3_)_2_, heterogeneous nanocatalyst 1 was synthesized. The structure of this nanocatalyst was confirmed by XRD, FT-IR, EDX, SEM and TGA techniques.

**Fig. 1:**
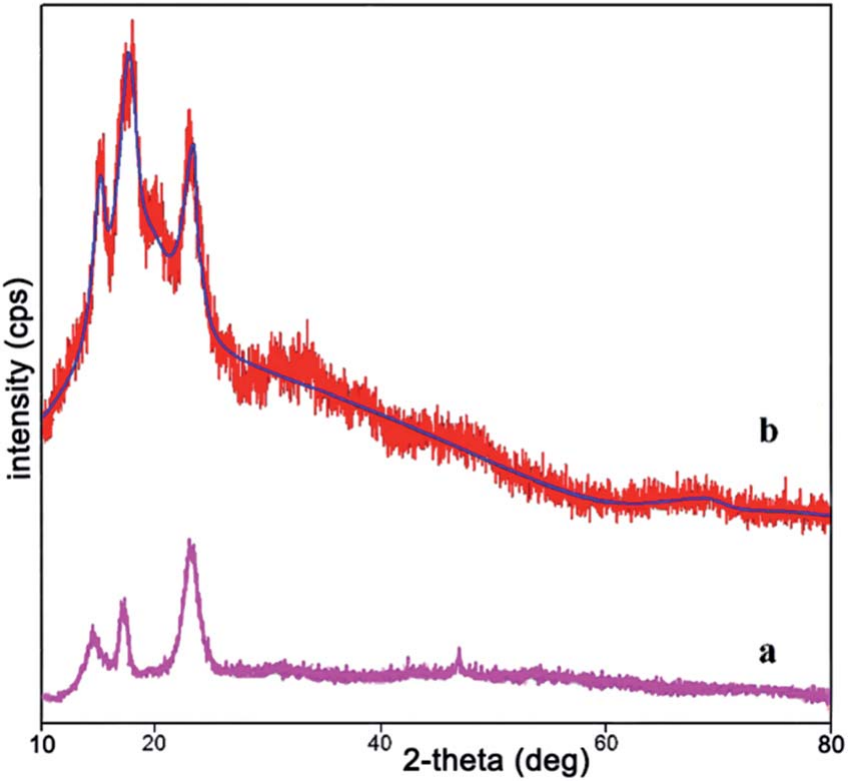
XRD pattern of (a) cellulose and (b) Ni^II^(BAPTE)(NO_3_)_2_-Cell nanocatalyst.

The XRD analysis was investigated for the structure of prepared nanocatalyst over the 2*θ* = 10–80° ranges (Fig. 1). Fig. 1a is related to XRD pattern of cellulose and Fig. 1b is related to Ni^II^(BAPTE)(NO_3_)_2_-Cell nanocatalyst. As shown in Fig. 1, cellulose appear to be composed of two parts, crystalline and amorphous. The peaks shown at 2*θ* ∼ 16.6 and 22.6 corresponding to the (002) and (001) confirm the crystalline portion of cellulose, and the peak at 18.8 corresponds to the amorphous region.^93^

**Fig. 2:**
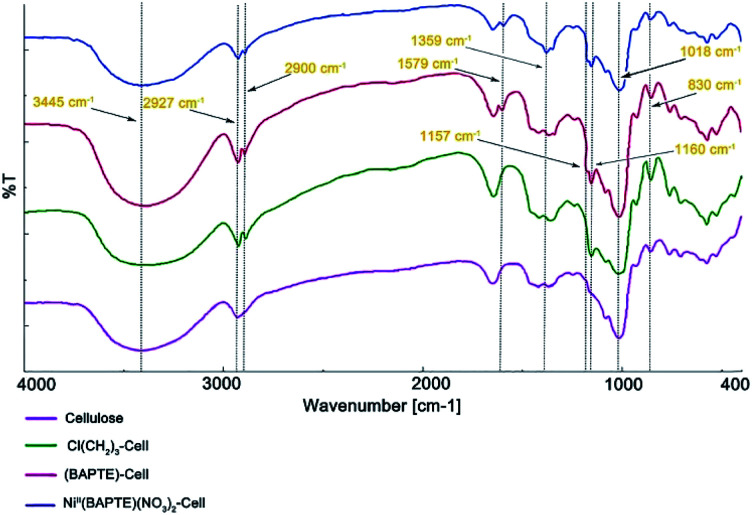
FT-IR spectra of (a) cellulose, (b) Cl(CH_2_)_3_-Cell, (c) BAPTE-Cell and (d) Ni^II^(BAPTE)(NO_3_)_2_-Cell.

The IR spectroscopy of cellulose, Cl(CH_2_)_3_-Cell, BAPTE-Cell and Ni^II^(BAPTE)(NO_3_)_2_-Cell are demonstrated in Fig. 2. The broad peaks in the range between 3000–3600 cm^−1^ in all spectra belong to the OH cellulose groups. In Fig. 2a, the peak shown at 2927 cm^−1^ characterizes the aliphatic C–H stretching vibration. The small peaks observed about 700–900 cm^−1^ are corresponding to C–OH. The peak of C–O–C is also specified in 1018 cm^−1^.^94^ In Fig. 2b, the new peaks in 2900 cm^−1^ and 1157 cm^−1^ can be attributed to C–H and Si–O–C stretching. N–H stretching band of BAPTE ligand in Fig. 2c and d overlap with hydroxyl groups of cellulose and the bending vibration of them was shown in 1579 cm^−1^.^95^ The peaks in 1160 cm^−1^ and 1359 cm^−1^ also is assigned to C–S and C–N stretching vibration, respectively. These observations and the presence of functional groups of expected catalysts in the final product confirm the structure of the catalyst.^96^

**Fig. 3:**
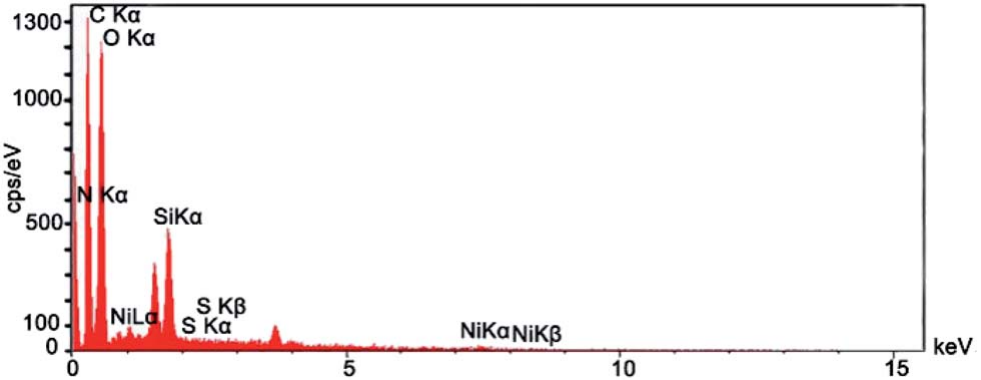
EDX spectrum of Ni^II^(BAPTE)(NO_3_)_2_-Cell nanocatalyst.

Fig. 3 shows the EDX spectrum of Ni^II^(BAPTE)(NO_3_)_2_-Cell which is used as an efficient technique to describe the components of the nanocatalyst. According to Fig. 3, EDS pattern clearly indicated the existence expected of the elemental composition of C, O, N, Si, Ni and S in the nanocatalyst structure.

**Fig. 4:**
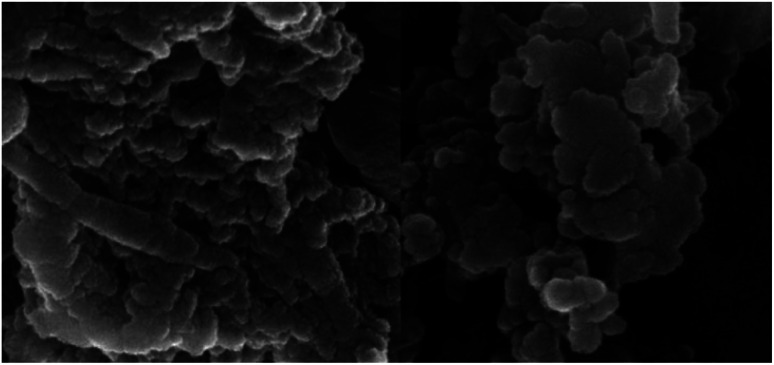
SEM image of nanocatalyst 1.

Identification of the size, morphological feature and shape of Ni^II^(BAPTE)(NO_3_)_2_-Cell nanocatalyst was characterized by scanning electron microscope (SEM) in Fig. 4. According to this image, the average particle size is 50 nm and the surface of the nanocatalyst is almost uniform.

**Fig. 5:**
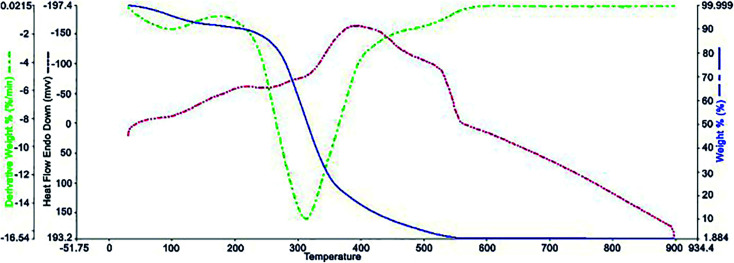
Thermogravimetric analysis curve of Ni^II^(BAPTE)(NO_3_)_2_-Cell nanocatalyst.

Fig. 5 shows the results of the TGA analysis at 25 to 900 °C. As can be seen, the Ni^II^(BAPTE)(NO_3_)_2_-Cell nanocatalyst was thermally stable at temperatures below 250 °C. The main sharp weight loss at this temperature (250–400 °C) is due to the thermal degradation of the carbohydrate support. The initial weight loss (∼2%) at temperatures below 100 °C is also related to the removal of the remaining alcoholic solvents and moisture from the nanocatalyst synthesis process.

**Scheme 2:**
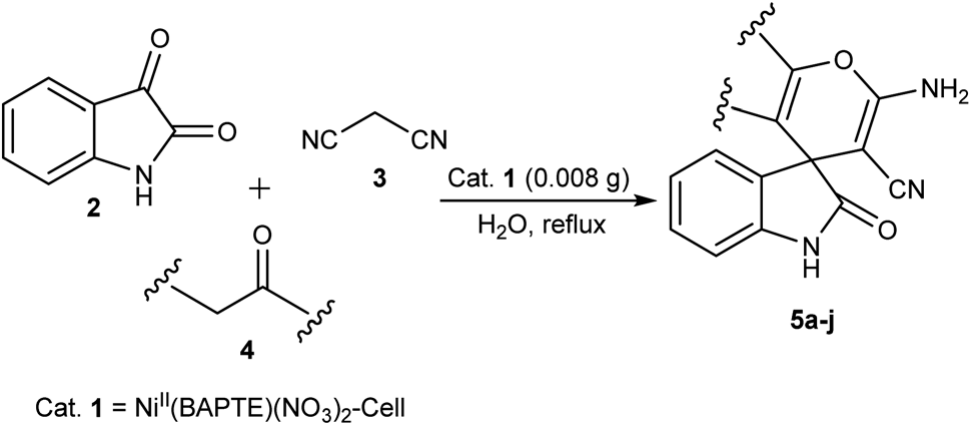
Synthesis of spirooxindole derivatives in the presence of nanocatalyst 1.

After characterization, to investigate the catalytic activity and efficiency of the newly designed catalyst, it was applied as a catalyst to synthesize spirooxindole derivatives by the three-component reaction of isatin 2, malononitrile 3 and compounds 4 (Scheme 2).

To find the optimal reaction conditions, the reaction between isatin, malononitrile and dimedone was selected as a model reaction, and the effect of catalyst amounts, temperature and solvent was investigated. The reaction did not progress well in the absence of the catalyst. The model reaction was performed in the presence of 0.002, 0.004, 0.008, and 0.010 g of catalyst 1 in water under reflux conditions. Next, the effect of the temperature was studied. The study showed the reaction to be affected by temperature and the best yield was obtained in refluxing water. Furthermore, the model reaction was performed by 0.008 g of Ni^II^(BAPTE)(NO_3_)_2_-Cell nanocatalyst in some solvents such as methanol, ethanol, acetonitrile, toluene and also under solvent-free conditions. As can be seen, considerable acceleration is observed chiefly in reactions performed in water as solvent. According to these results, the use of 0.008 g of Ni^II^(BAPTE)(NO_3_)_2_-Cell as catalyst in water under reflux conditions would be the best of choice (Table 1). In the following, we employed the optimum conditions for the synthesis of some spirooxindole derivatives using different reactive methylene compounds 5 (Table 2).

**Table 1:** Optimization of the reaction condition of synthesis of product 5a^a^

Entry	Catalyst 1 (g)	Solvent	Temp. (°C)	Yield^b^ (%)
1	—	H_2_O	Reflux	10
2	0.002	H_2_O	Reflux	50
3	0.004	H_2_O	Reflux	65
4	0.008	H_2_O	Reflux	95
5	0.010	H_2_O	Reflux	90
6	0.008	H_2_O	r.t.	60
7	0.008	H_2_O	50	65
8	0.008	H_2_O	80	50
9	0.008	MeOH	Reflux	60
10	0.008	EtOH	Reflux	65
11	0.008	CH_3_CN	Reflux	55
12	0.008	Toluene	Reflux	60
13	0.008	—	100	60

a Reaction conditions: isatin (1 mmol), malononitrile (1 mmol) and dimedone (1 mmol), catalyst 1, time: 20 min.

b Isolated yields.

**Table 2:** Ni^II^(BAPTE)(NO_3_)_2_-Cell catalyzed synthesis of spirooxindole derivatives

Entry	Compound 4	Product	Yield^a^ (%)
5a	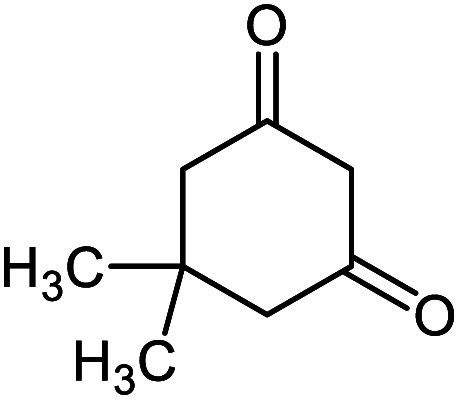	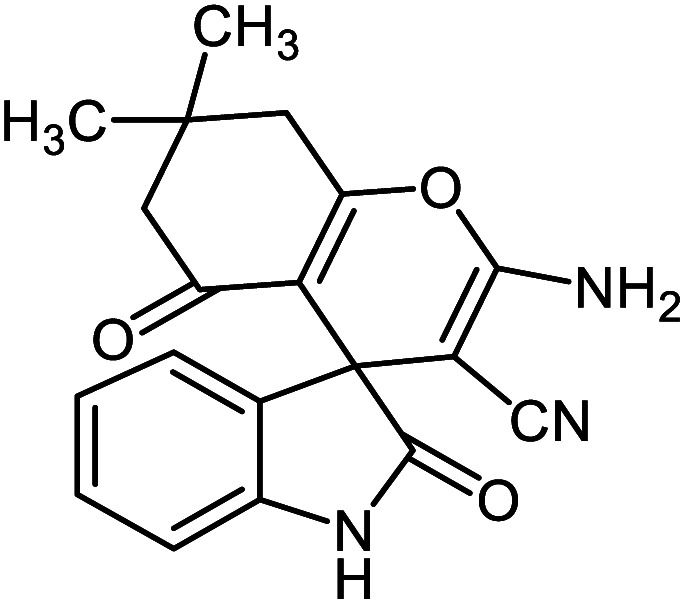	95 (ref. 97)
5b	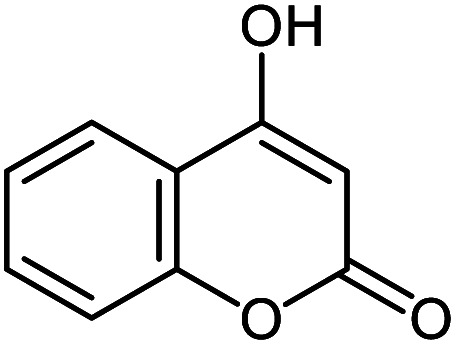	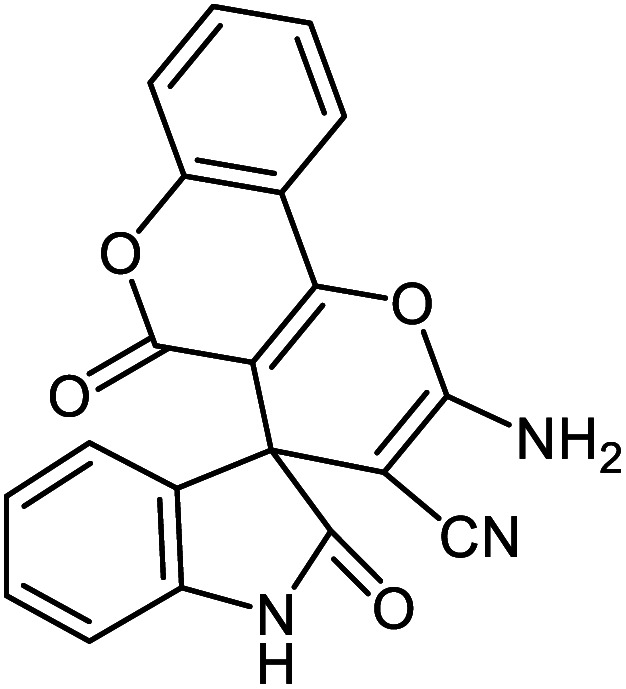	92 (ref. 97)
5c	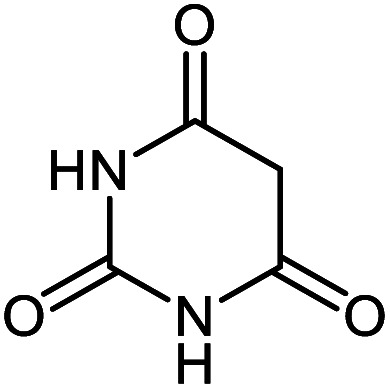	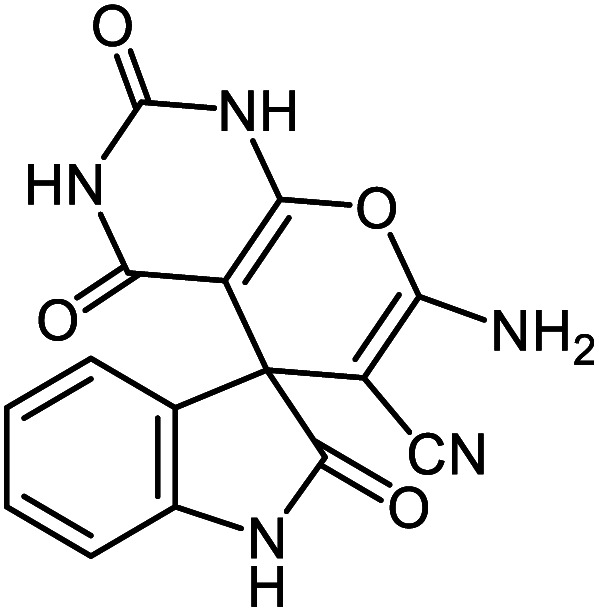	95 (ref. 98)
5d	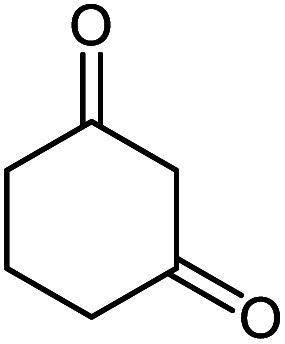	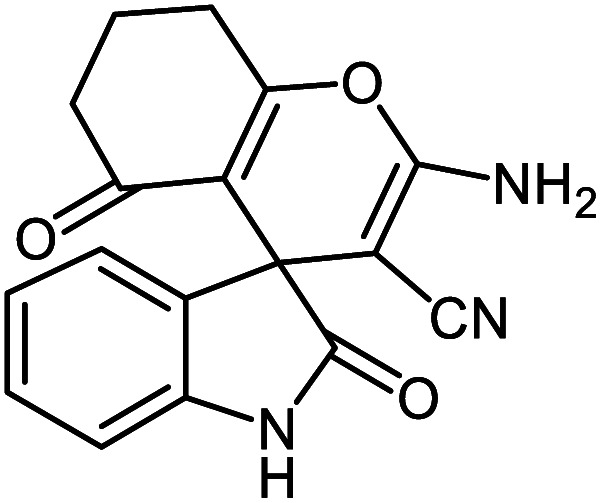	96 (ref. 97)
5e	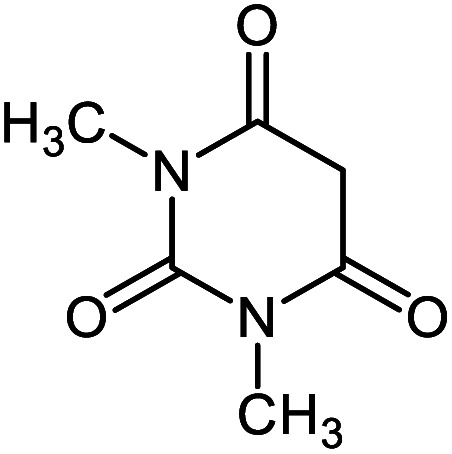	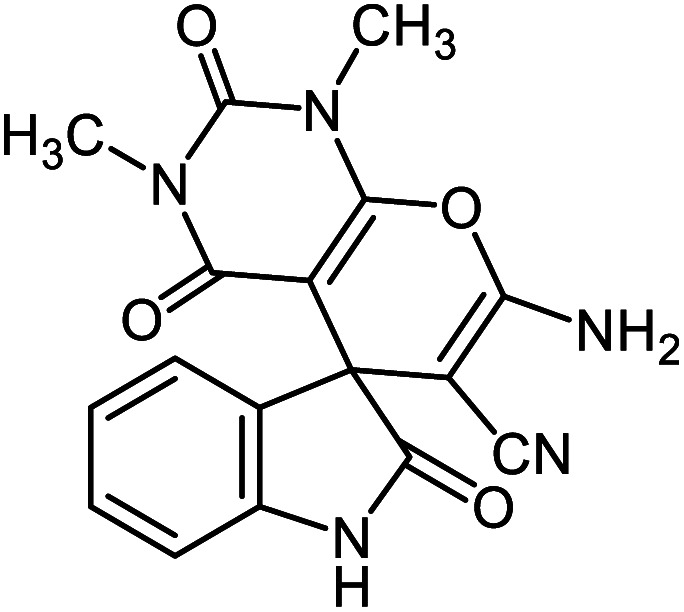	87 (ref. 98)
5f	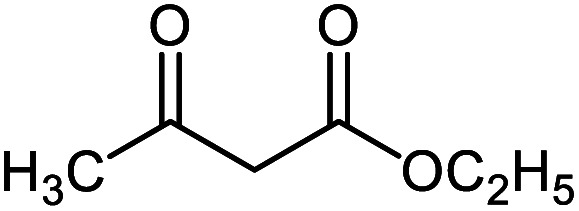	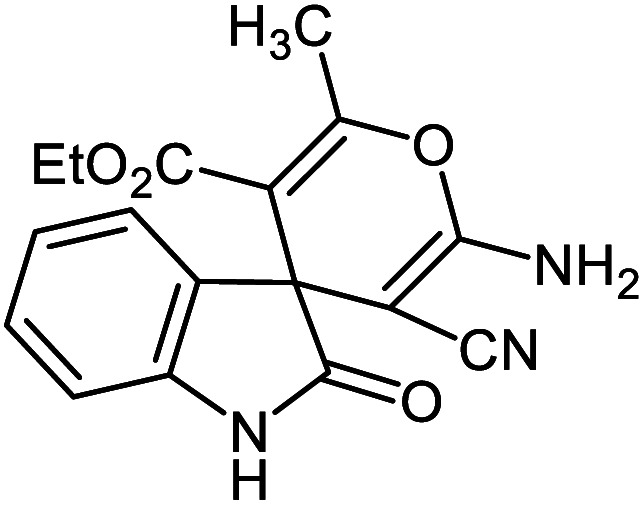	85 (ref. 99)
5g	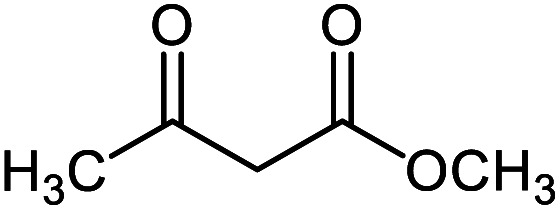	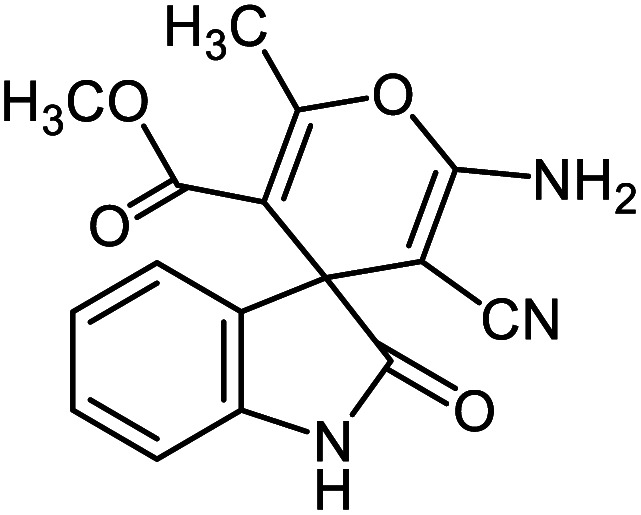	87 (ref. 100)
5h	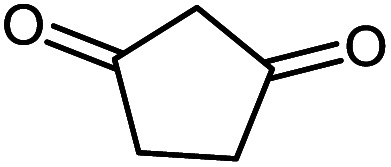	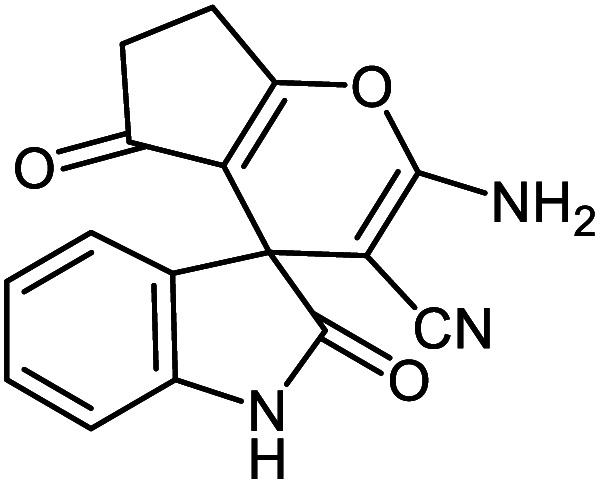	80 (ref. 97)
5i	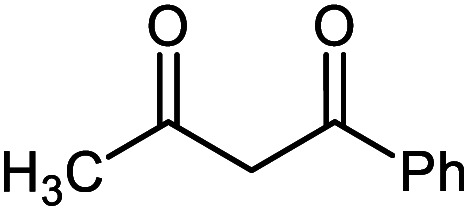	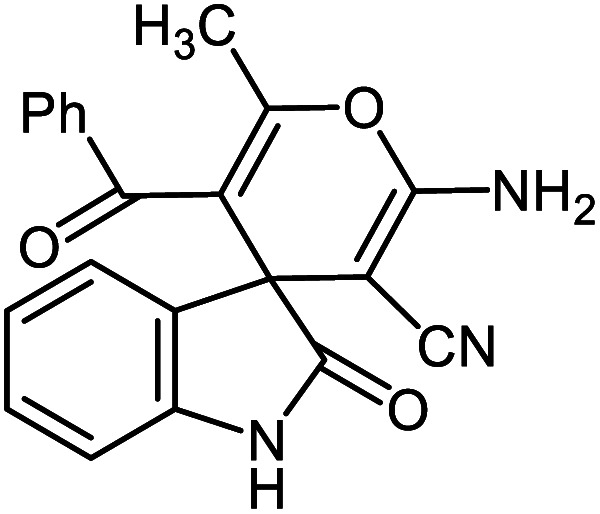	80 (ref. 99)
5j	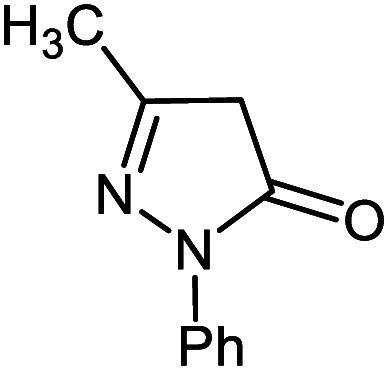	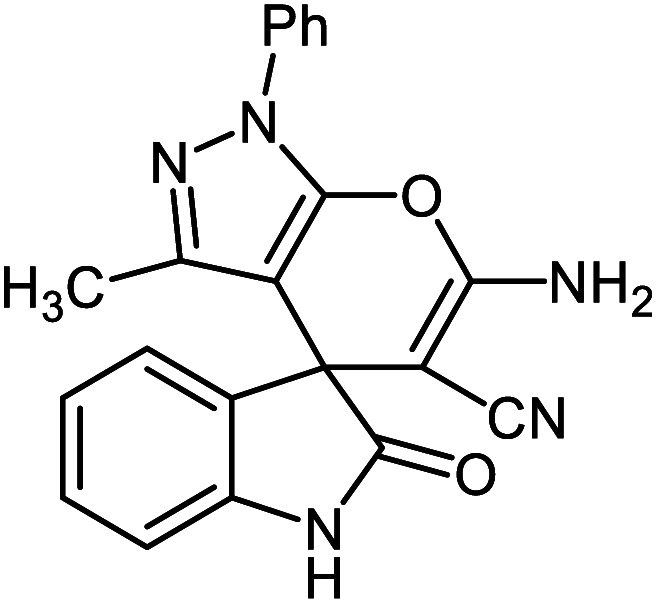	75 (ref. 101)

a Isolated yields.

**Scheme 3:**
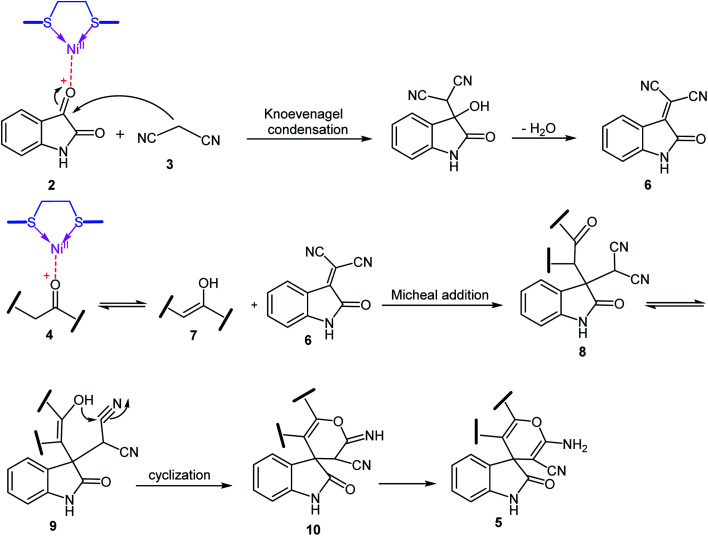
The probable mechanism for the synthesis of spirooxindole derivatives in the presence of Ni^II^(BAPTE)(NO_3_)_2_-Cell.

A possible mechanism for Ni^II^(BAPTE)(NO_3_)_2_-Cell-catalyzed synthesis of spirooxindole derivatives is shown in Scheme 3. Initially, malononitrile attacks to activated isatin to form intermediate 6. The reactive methylene compound 4 is converted to enol 7 in the presence of the catalyst and is thus activated. Then 7 attacks to 6 (Michael addition) to produce intermediate 8. Adduct 8 is converted to the enol form 9. After intramolecular cyclization of 9 followed by a tautomerization, the desired product 5 was obtained.

In another study, a filtration test was performed in order to investigate whether the catalyst operates in a homogeneous or heterogeneous manner. We performed the leaching study on the model reaction under optimum conditions, so after 10 min, EtOH (1 mL) was added and the obtained mixture was filtered to separate the catalyst. The reaction was allowed to continue and. In this regard, no considerable reaction progress was observed indicating that the active catalytic centers were not washed from the support during the reaction and the catalyst most likely worked in a heterogeneous manner.

**Fig. 6:**
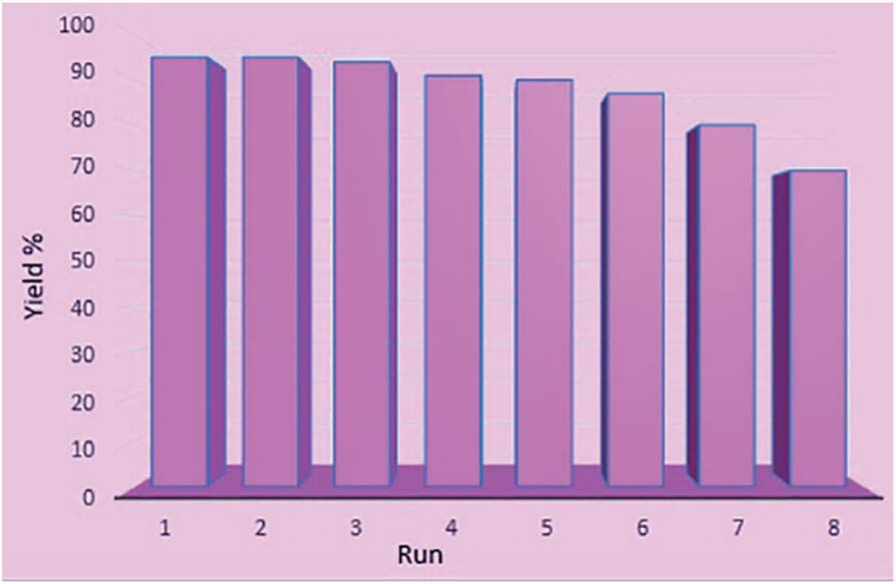
Reusability of Ni^II^(BAPTE)(NO_3_)_2_-Cell nanocatalyst in the reaction of isatin (1 mmol), malononitrile (1 mmol) and dimedone (1 mmol).

Subsequently, recovery and reuse of the catalyst were also investigated in the reaction of isatin, malononitrile and dimedone under standard reaction conditions. In this way, after the completion of the reaction, boiling ethanol was added, the catalyst was separated, washed with absolute ethanol and distilled water, and after drying, it was reused in the same reaction. The results showed that in eight reuses, the catalyst produced products with good efficiency without significant reduction in performance (Fig. 6).

**Fig. 7:**
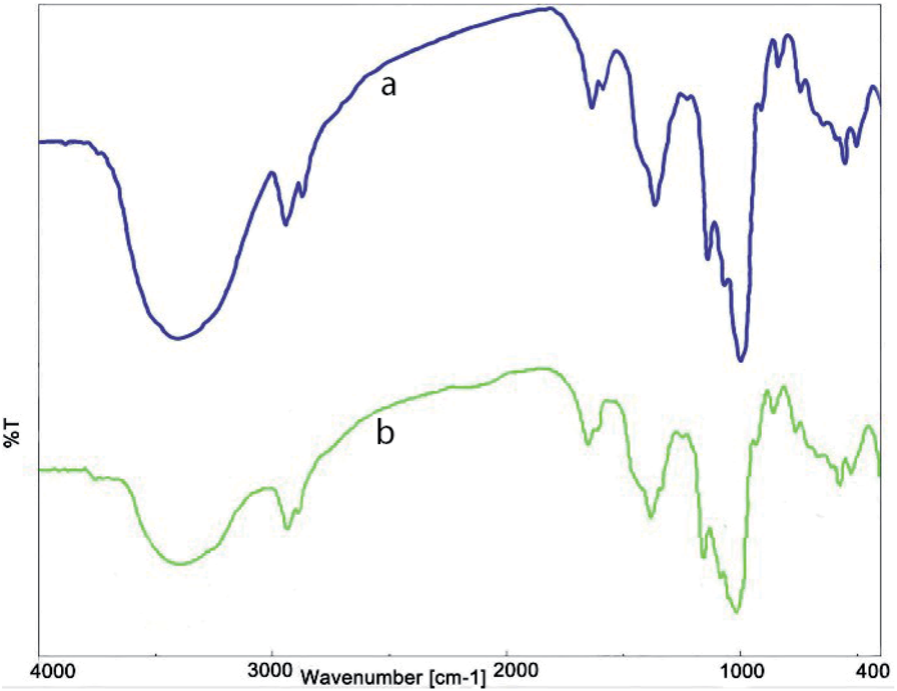
FT-IR spectrum of (a) fresh Ni^II^(BAPTE)(NO_3_)_2_-Cell and (b) reused Ni^II^(BAPTE)(NO_3_)_2_-Cell nanocatalyst.

In order to confirm the stability of the nanocatalyst during the reaction process, FT-IR analysis was performed on the recycled catalyst (Fig. 7). The presence of the specified index peaks, and their similarity to the FT-IR spectrum of fresh nanocatalyst, proves that the structure of the nanocatalyst, after eight reuses in the model reaction, remains almost unchanged.

## Conclusions

In summary, we have developed a heterogeneous nanocatalyst with a natural support and used it for the preparation of spirooxindole derivatives. Cellulose, which was applied as a suitable support for this purpose, not only causes good dispersion of Ni^II^(BAPTE)(NO_3_)_2_-Cell in the reaction mixture, but also show high catalytic activity in the synthesis of spirooxindole derivatives in a very short time. This new catalytic system demonstrated the advantages of environmentally benign character, easily separation, non-toxicity, mild reaction conditions, short reaction times as well as good reusability.

## Conflicts of interest

There are no conflicts to declare.

## Supplementary Material

RA-012-D1RA08182A-s001
