# Supplementary material for: Novel cellulose supported 1,2-bis(4-aminophenylthio)ethane Ni(ii) complex (NiII(BAPTE)(NO3)2-Cell) as an efficient nanocatalyst for the synthesis of spirooxindole derivatives
Source: RSC Adv. 2022 Jan 27;12(6):3584–92. doi: 10.1039/d1ra08182a (PMC8979259; doi:10.1039/d1ra08182a)

Electronic Supplementary Material (ESI) for RSC Advances.
This journal is © The Royal Society of Chemistry 2022

Supporting information

Novel cellulose supported 1,2-bis(4-aminophenylthio)ethane Ni (II) complex ($Ni^{II}(BAPTE)(NO_3)_2$-Cell) as an efficient nanocatalyst for the synthesis of spirooxindole derivatives

Raziyeh Keshavarz and Mahnaz Farahi*

Department of Chemistry, Yasouj University, P. O. Box 353, Yasouj 75918-74831, Iran

*Corresponding author: E-mail: farahimb@yu.ac.ir

^{1}H NMR spectrum of 5a

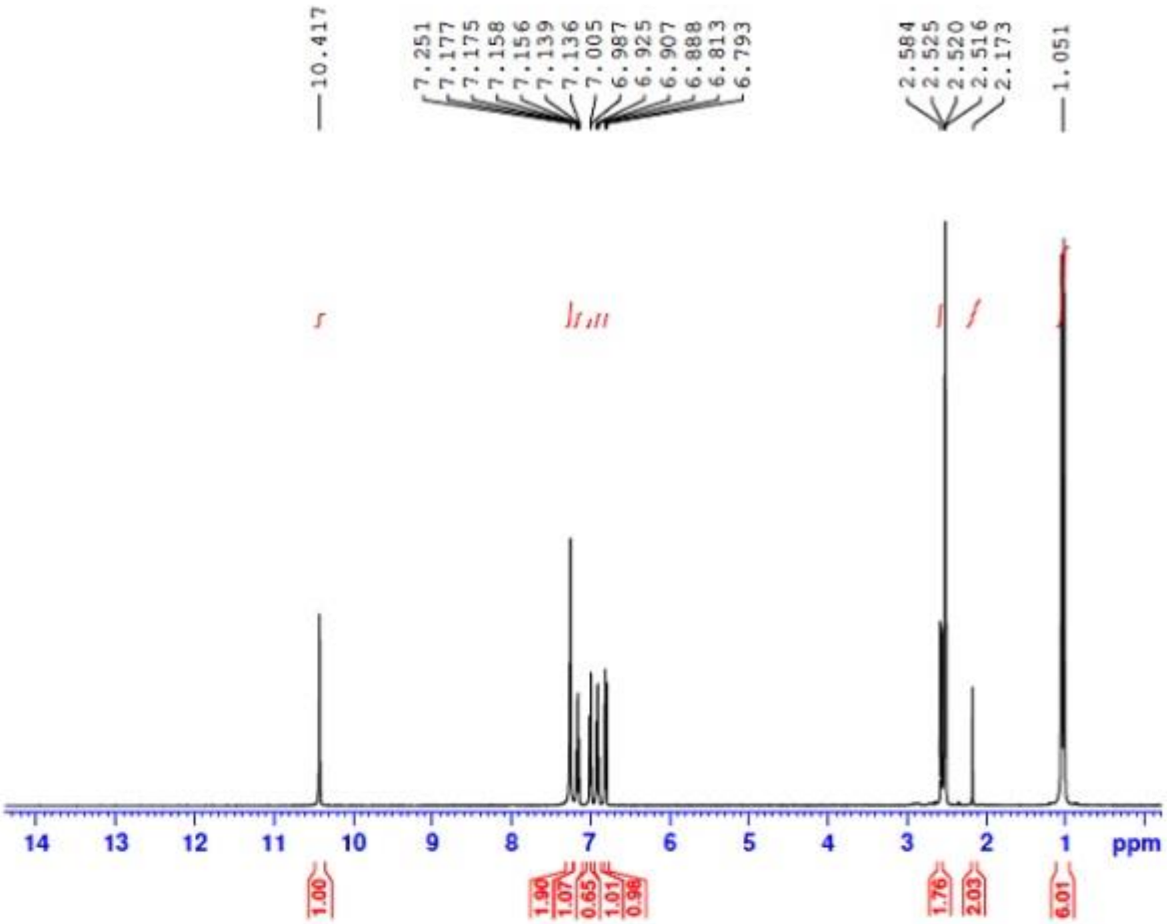

^{13}C NMR spectrum of 5a

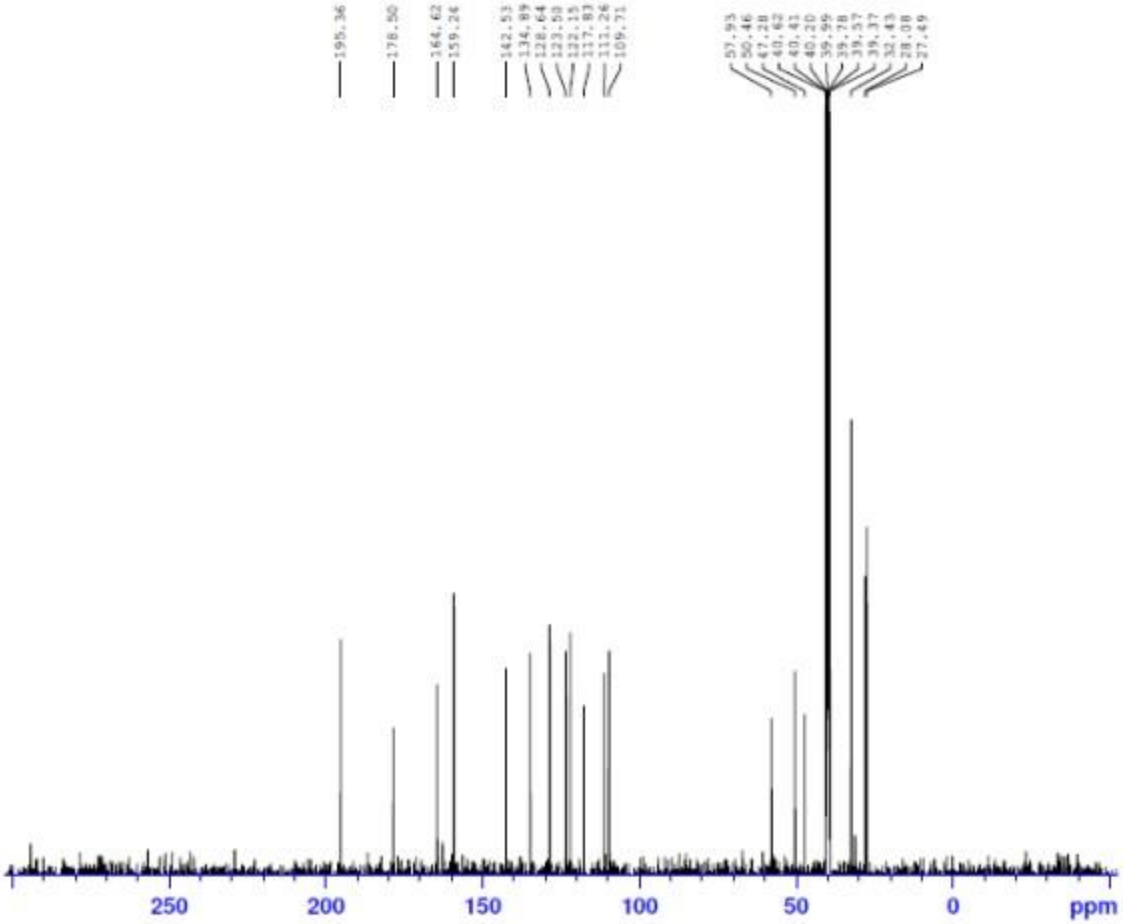

^{1}H NMR spectrum of 5b

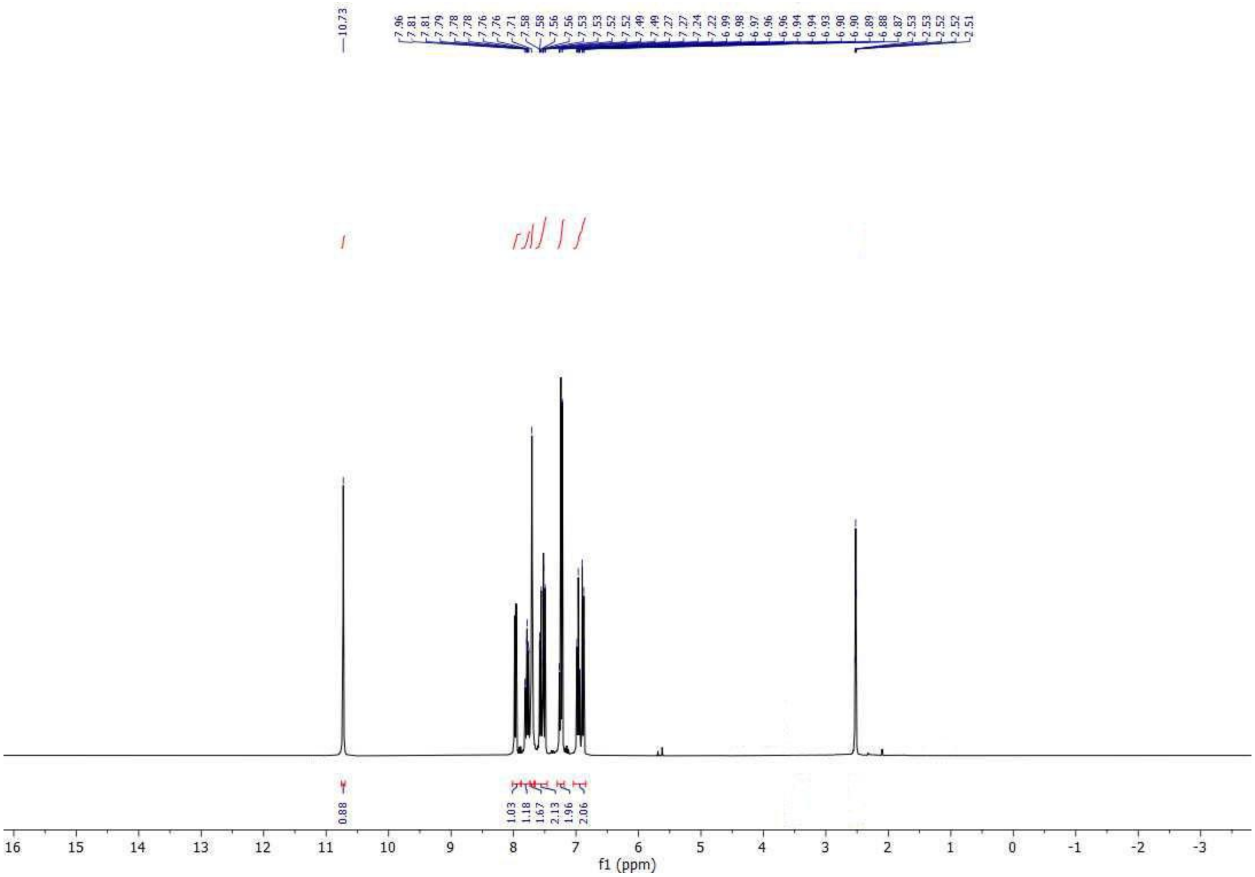

^{13}C NMR spectrum of 5b

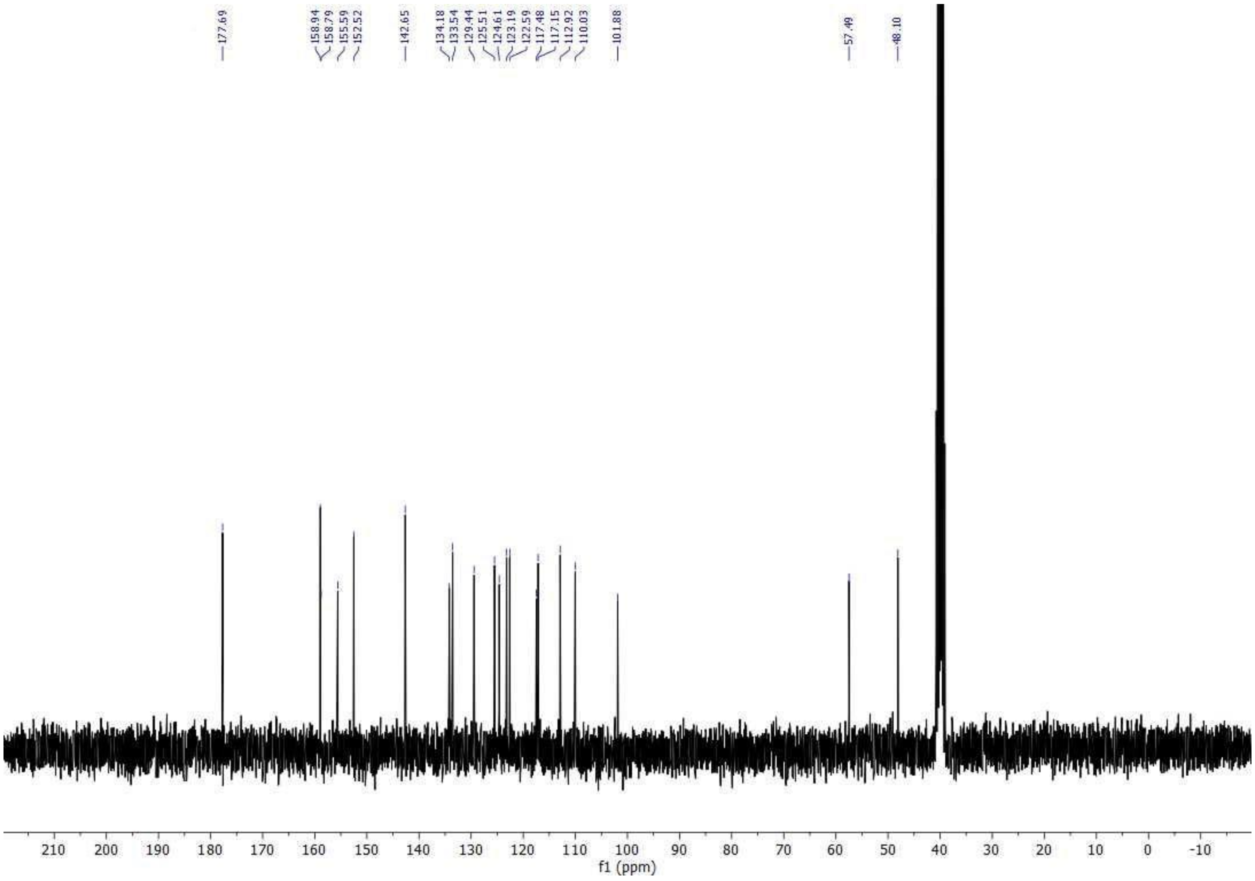

^1H NMR spectrum of 5c

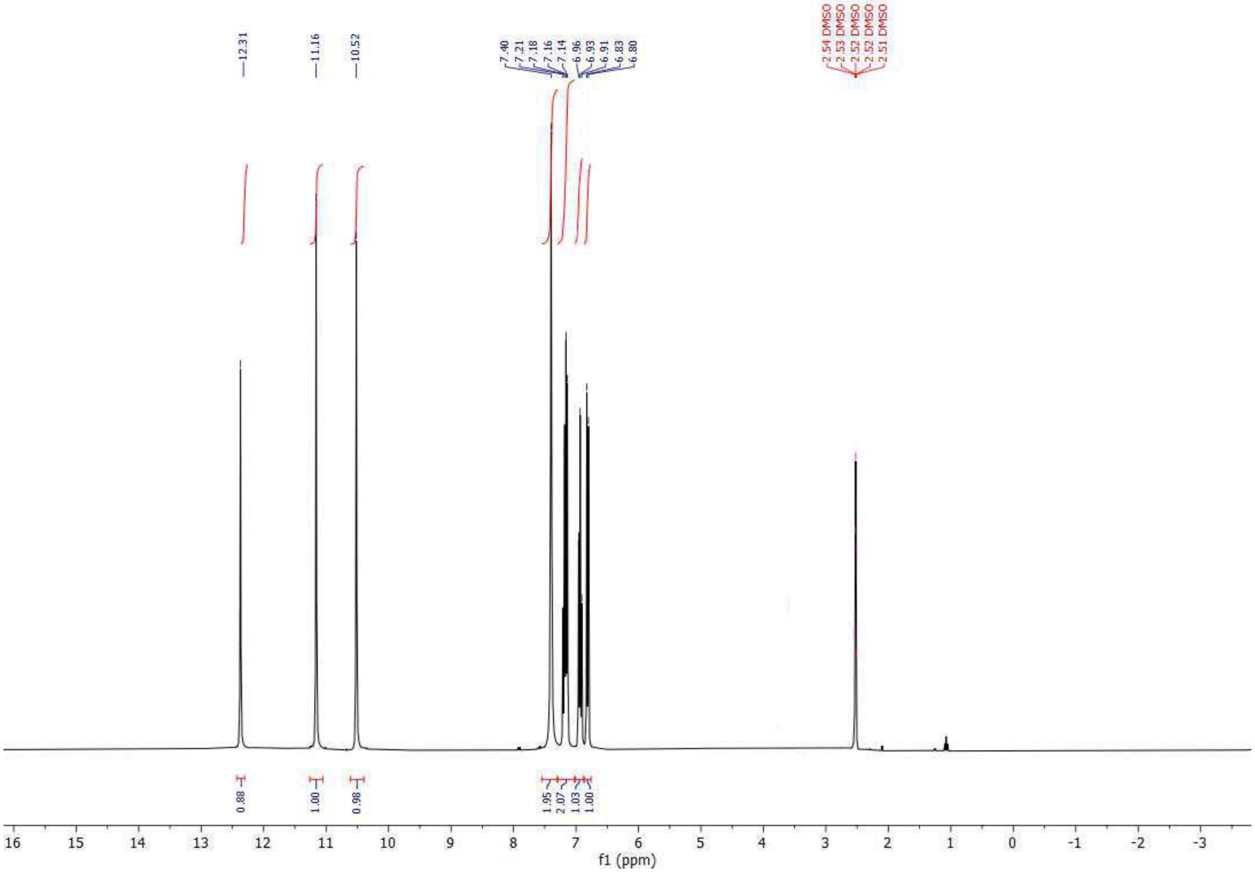

^{13}C NMR spectrum of 5c

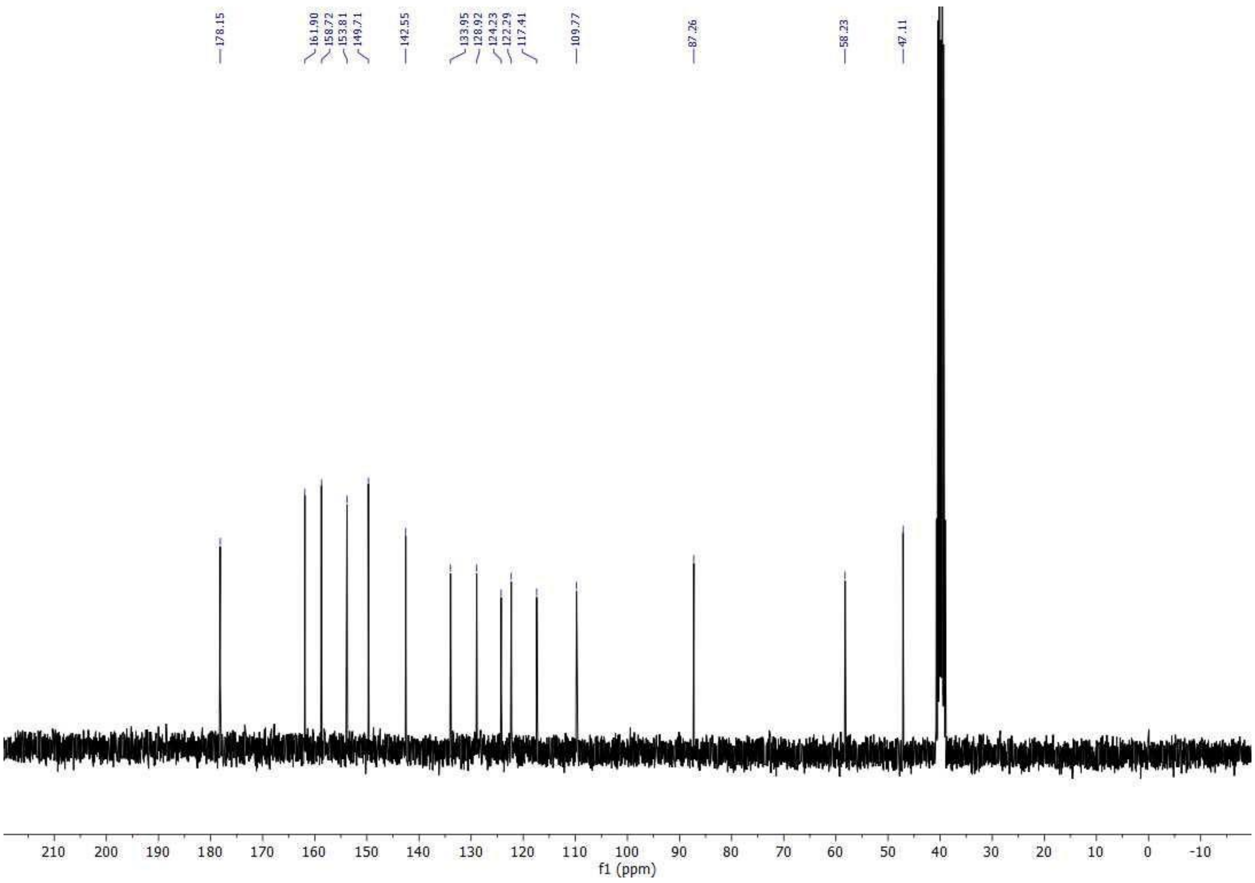

^{1}H NMR spectrum of 5d

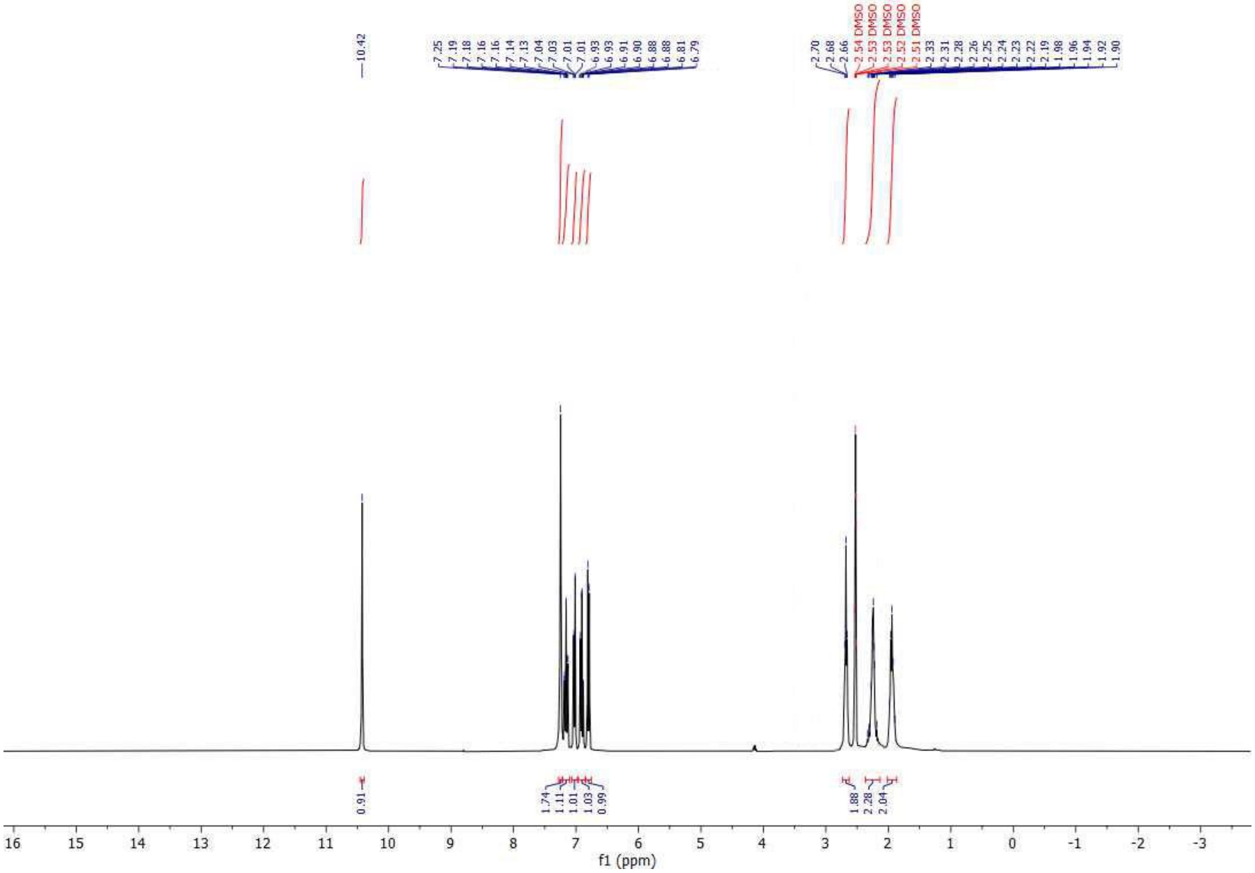

^{13}C NMR spectrum of 5d

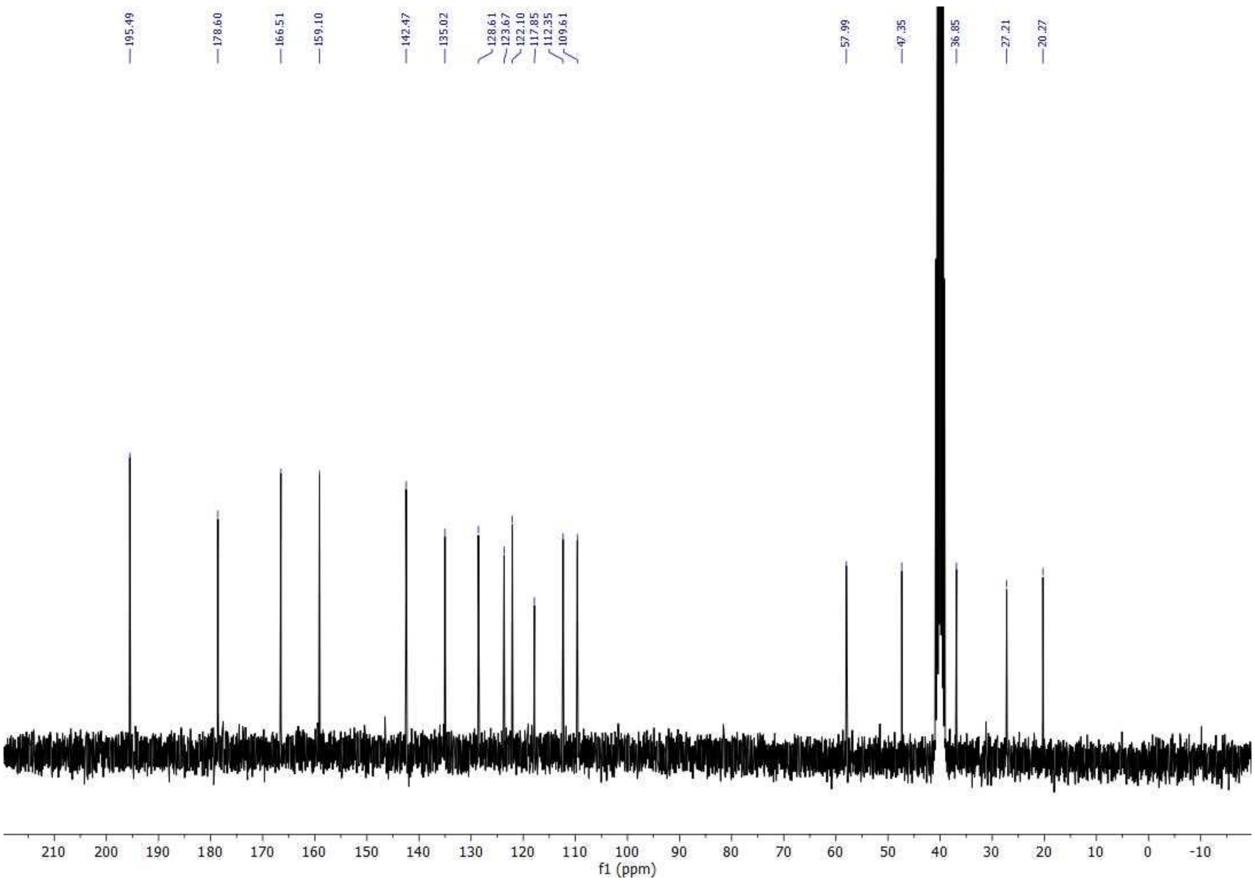

^{1}H NMR spectrum of 5e

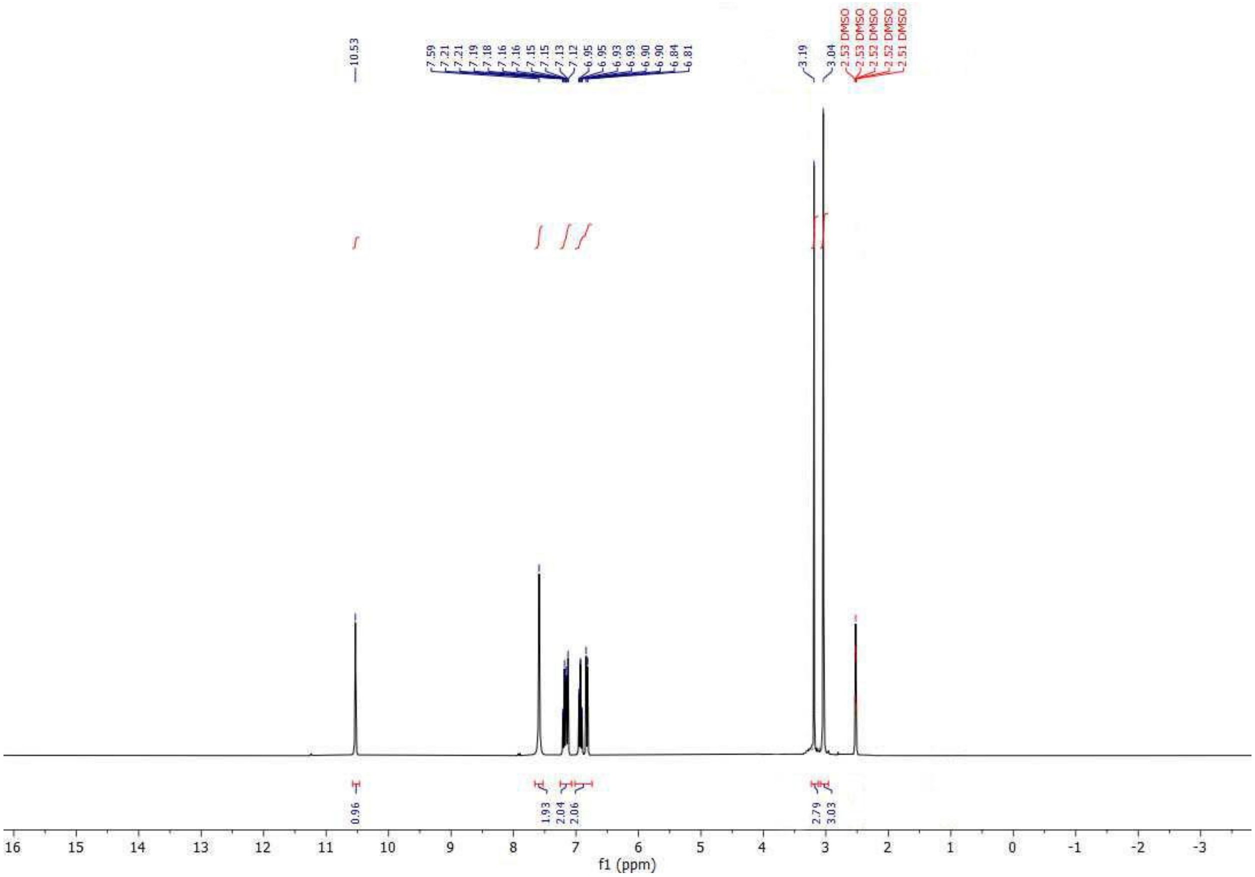

^{13}C NMR spectrum of 5e

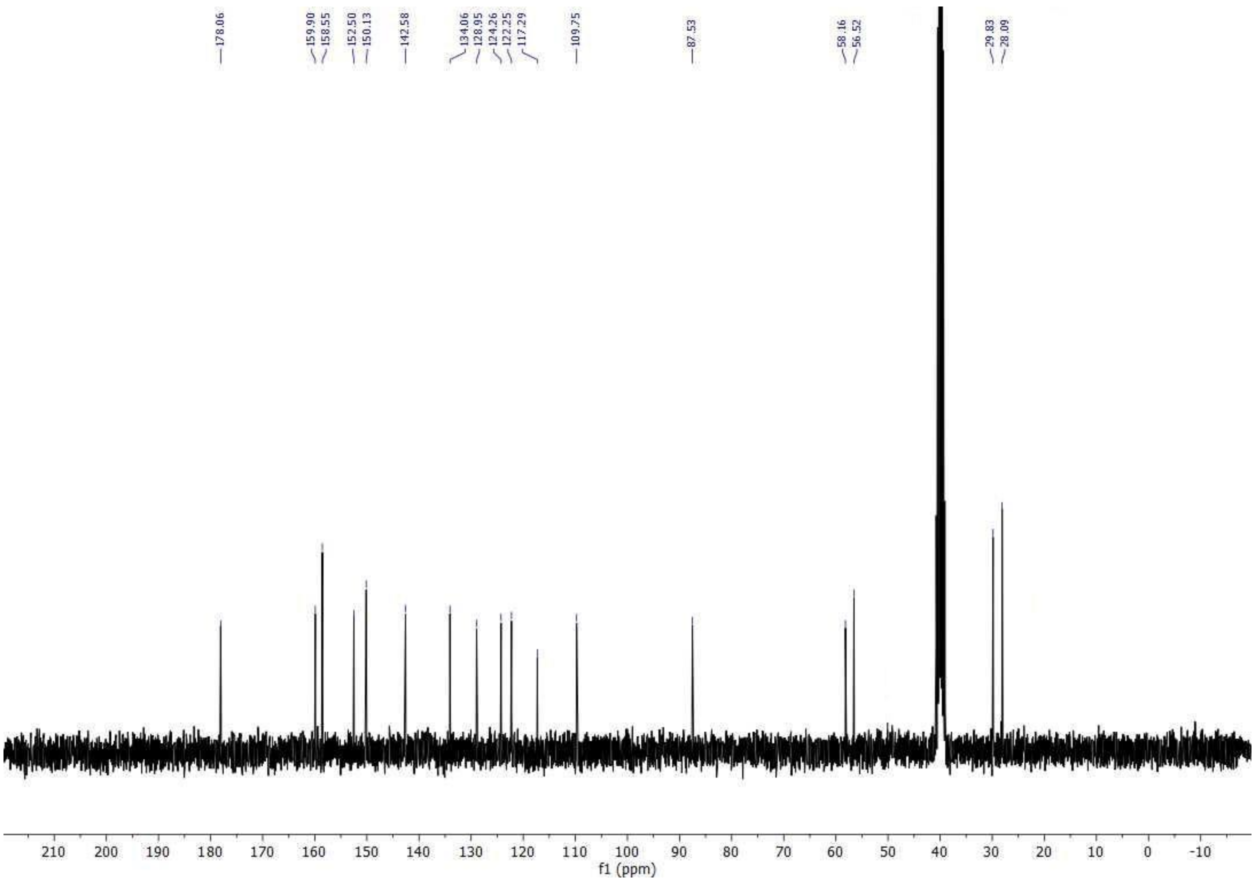

^1H NMR spectrum of 5f

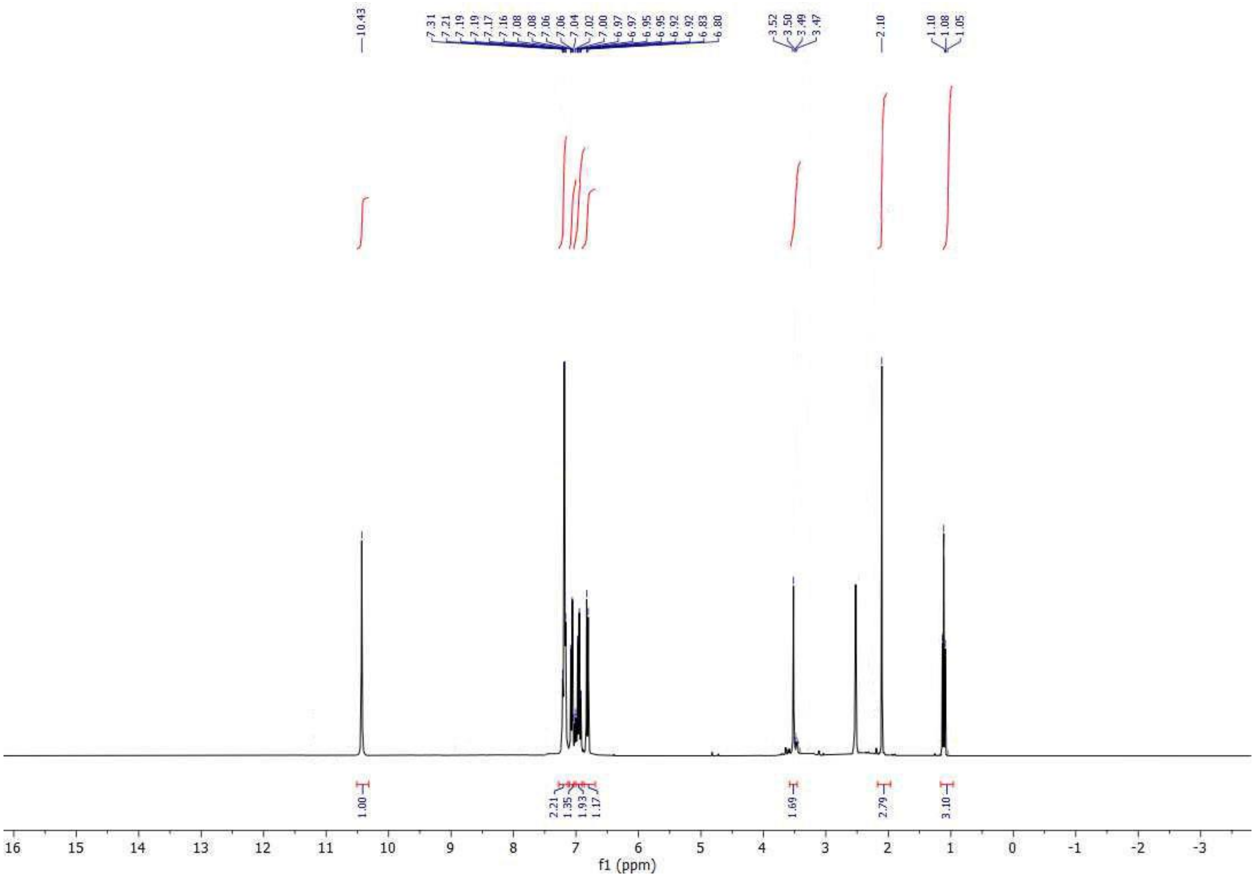

^{13}C NMR spectrum of 5f

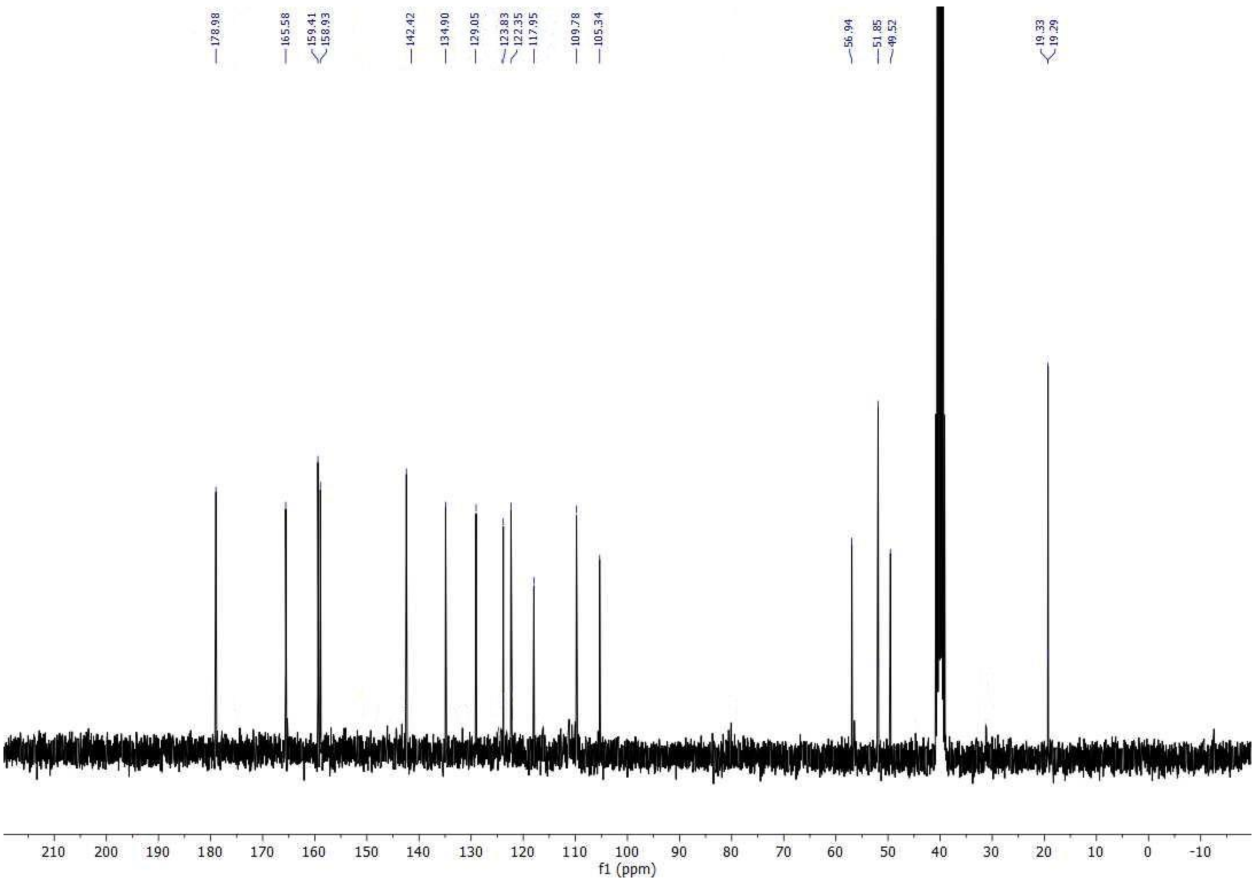

^{1}H NMR spectrum of 5g

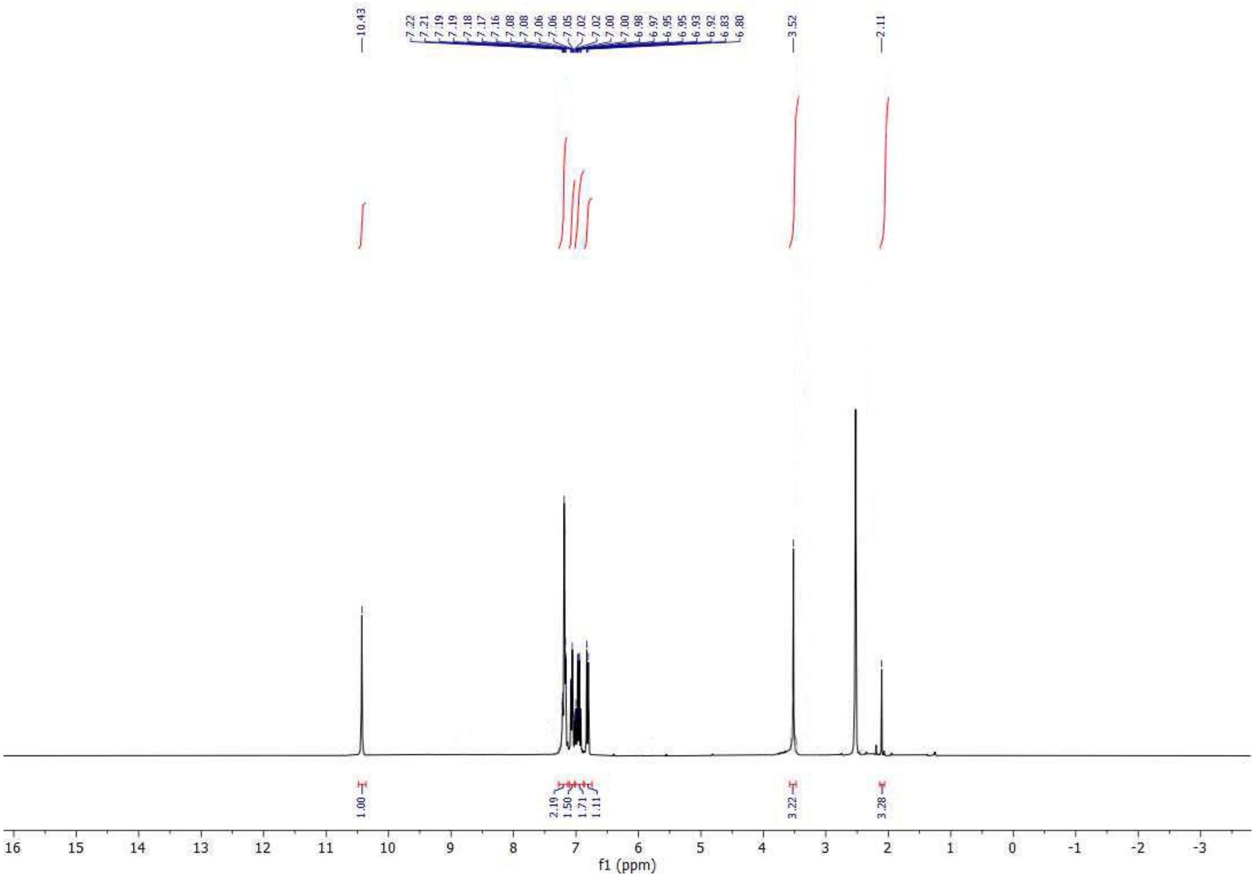

^{13}C NMR spectrum of 5g

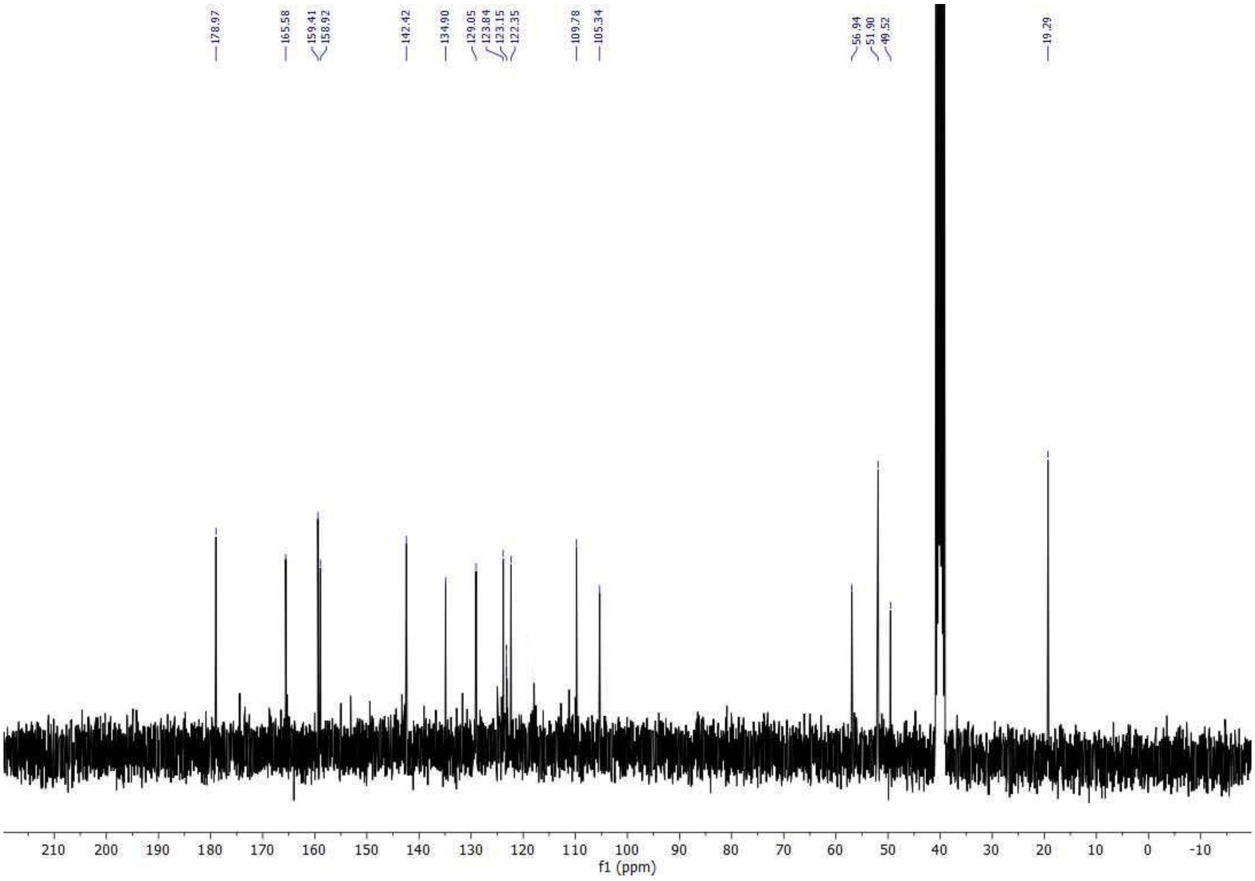

^{1}H NMR spectrum of 5h

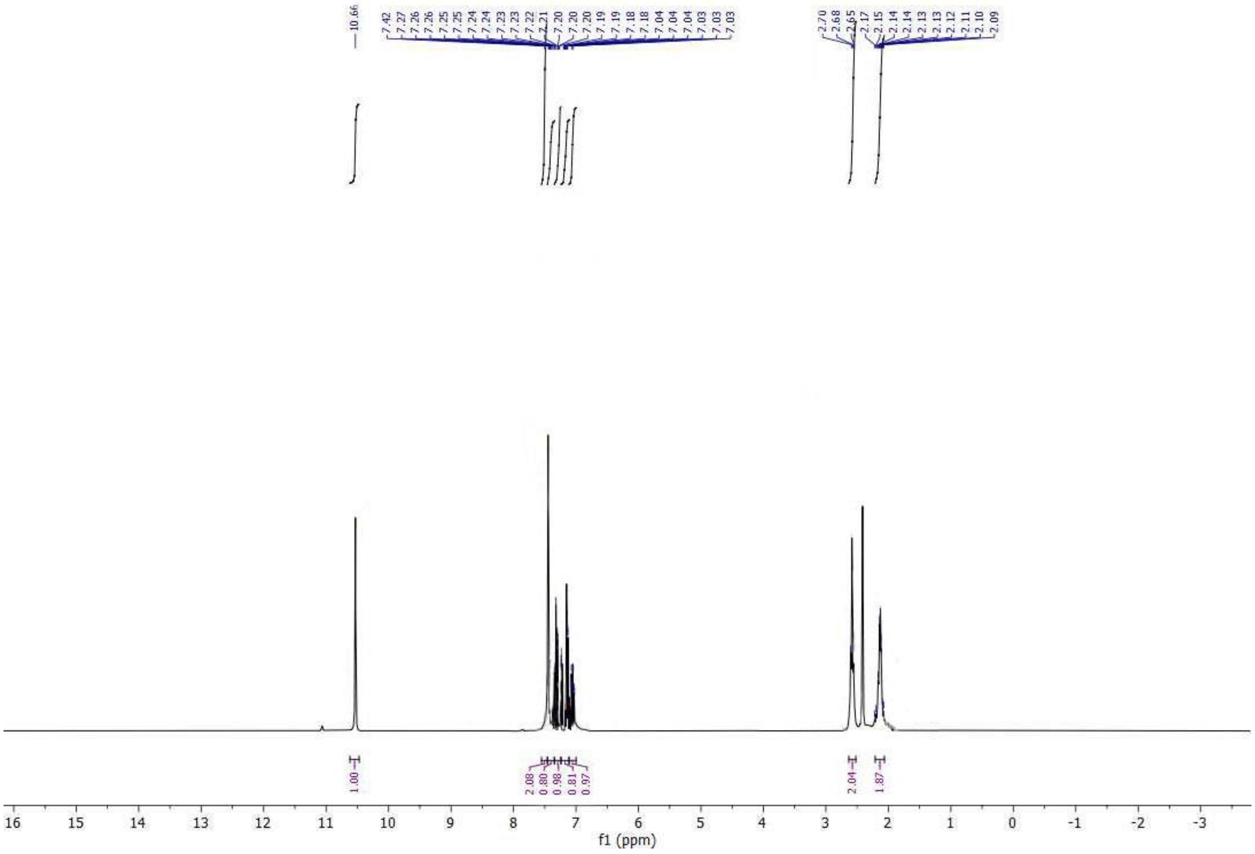

^{13}C NMR spectrum of 5h

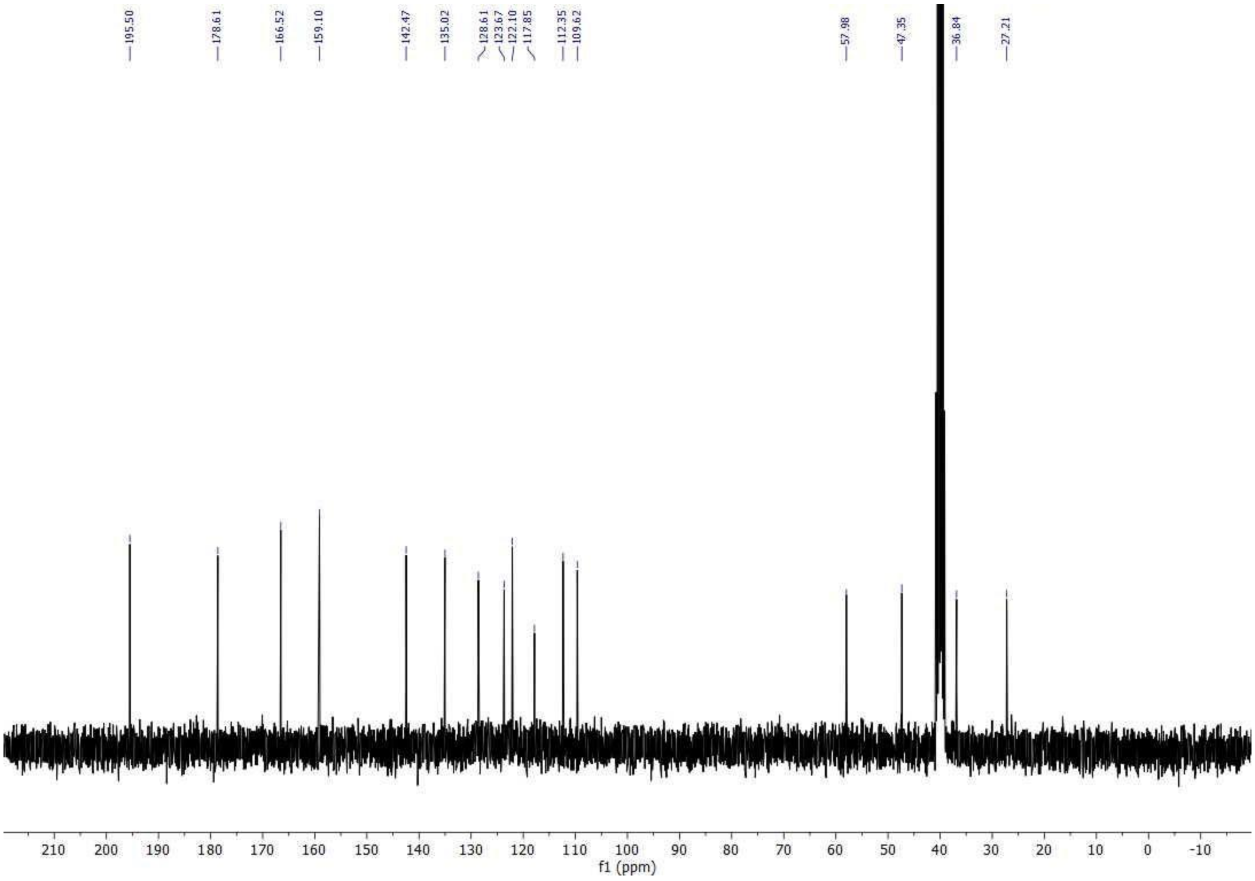

^{1}H NMR spectrum of 5i

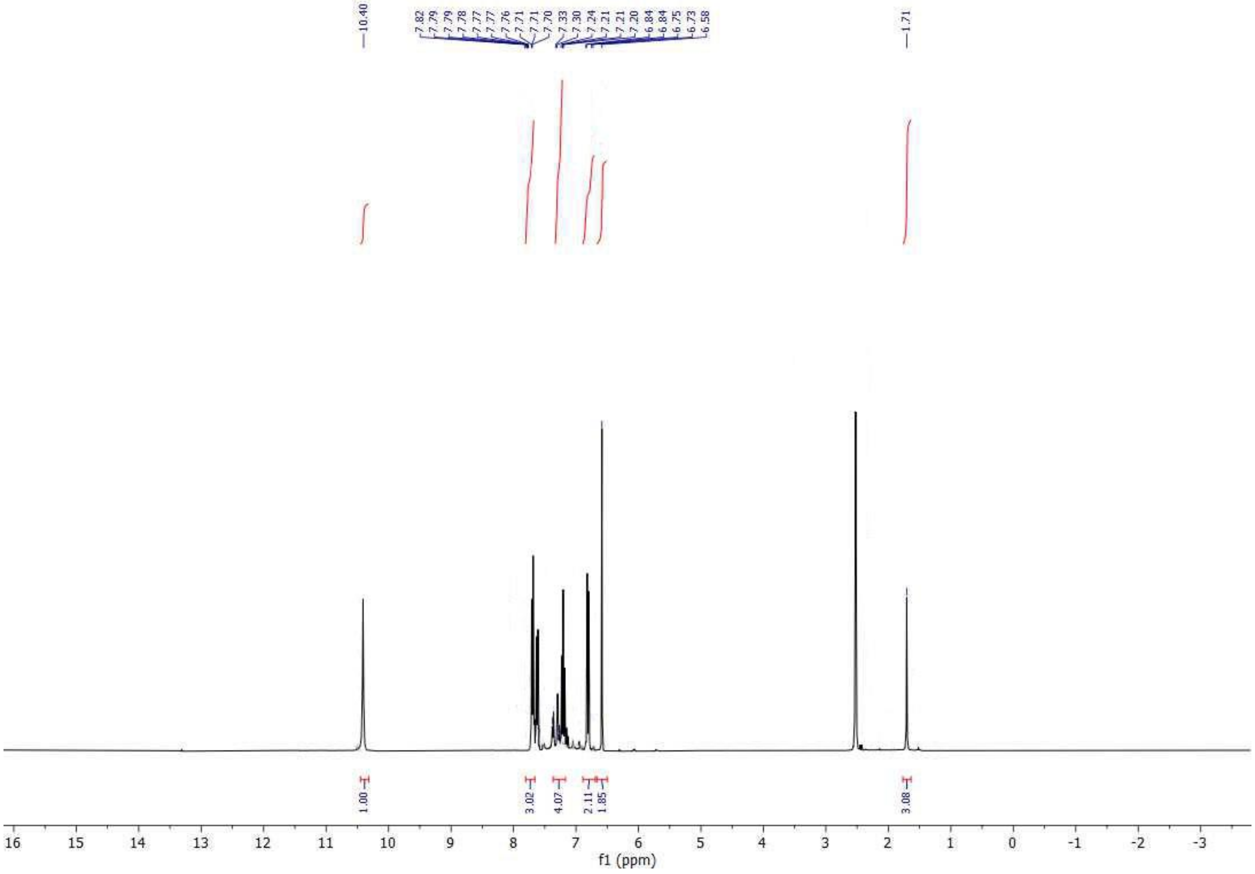

^{13}C NMR spectrum of 5i

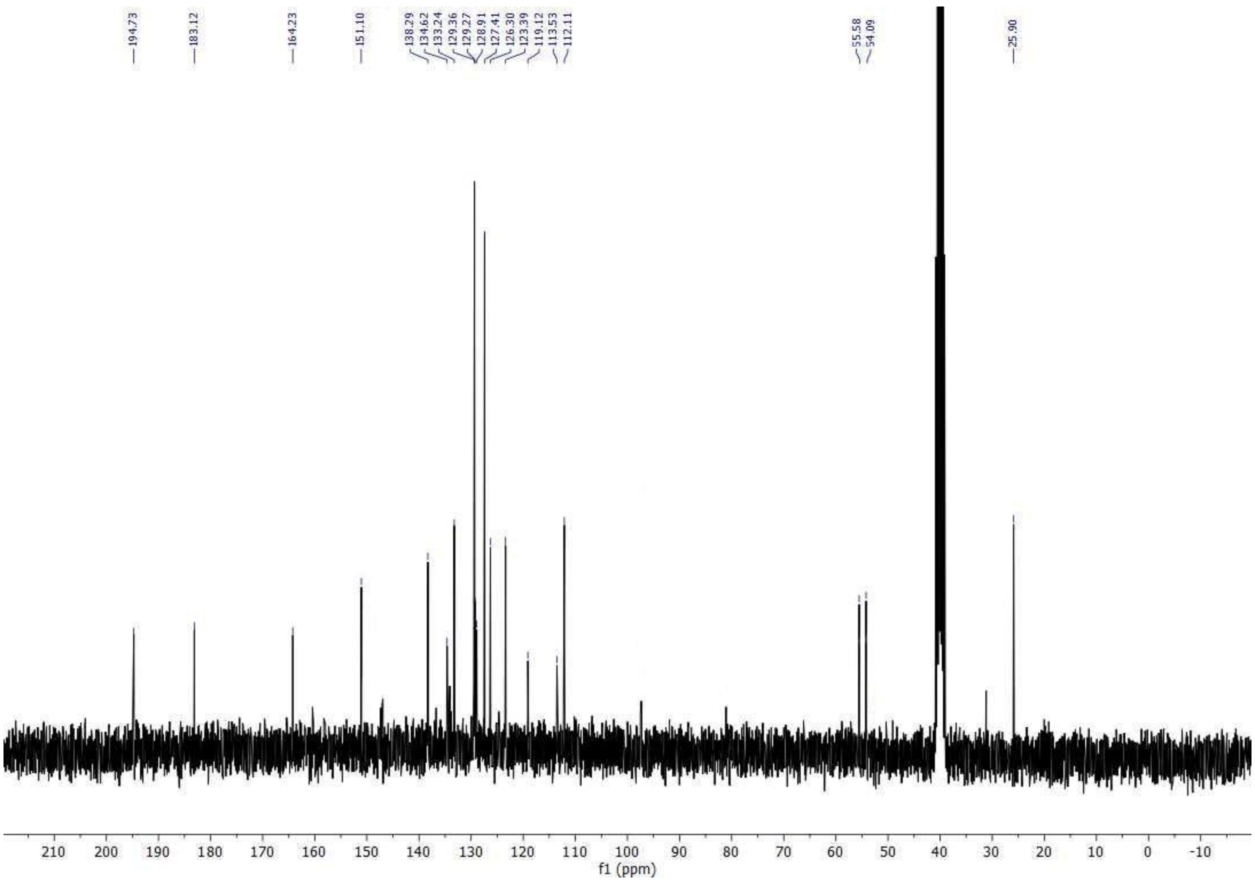

^{1}H NMR spectrum of 5j

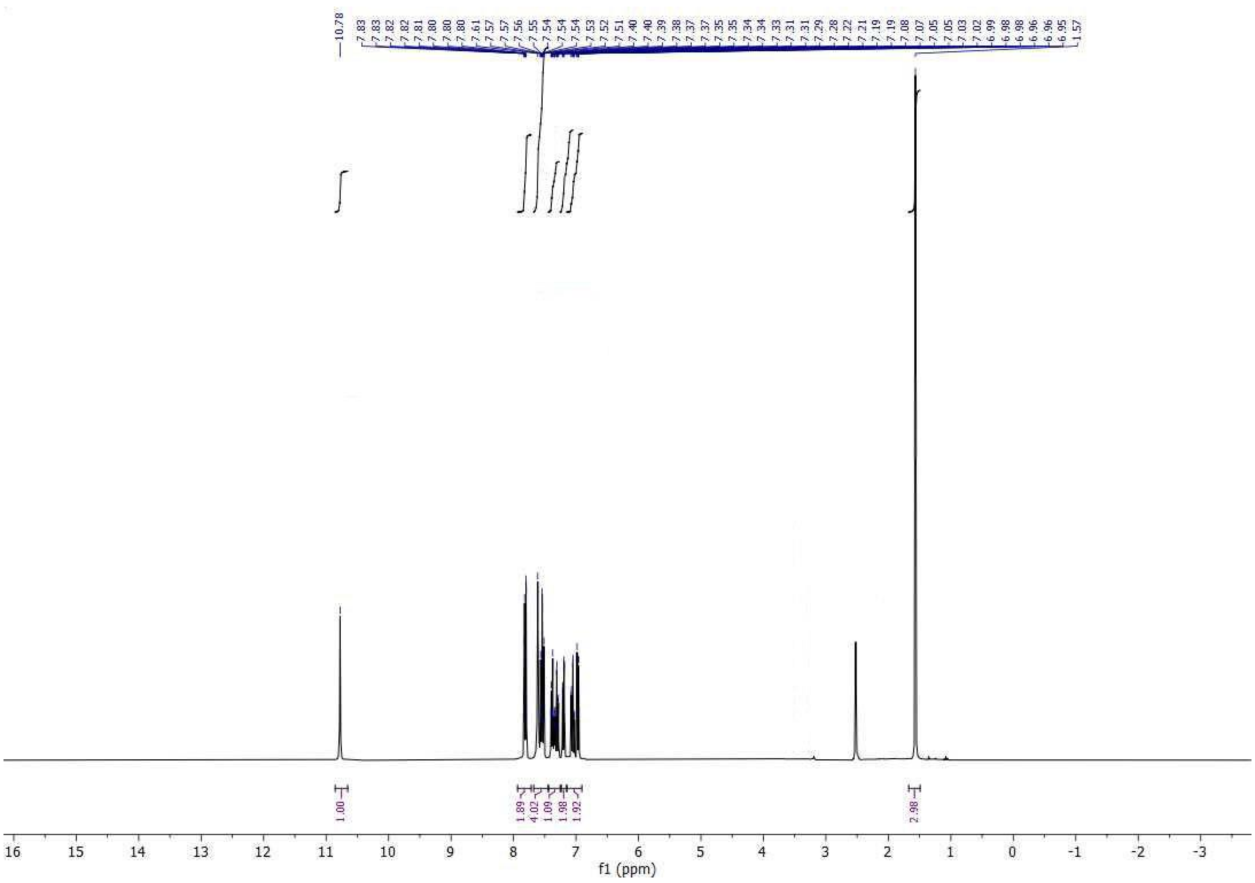

^{13}C NMR spectrum of 5j

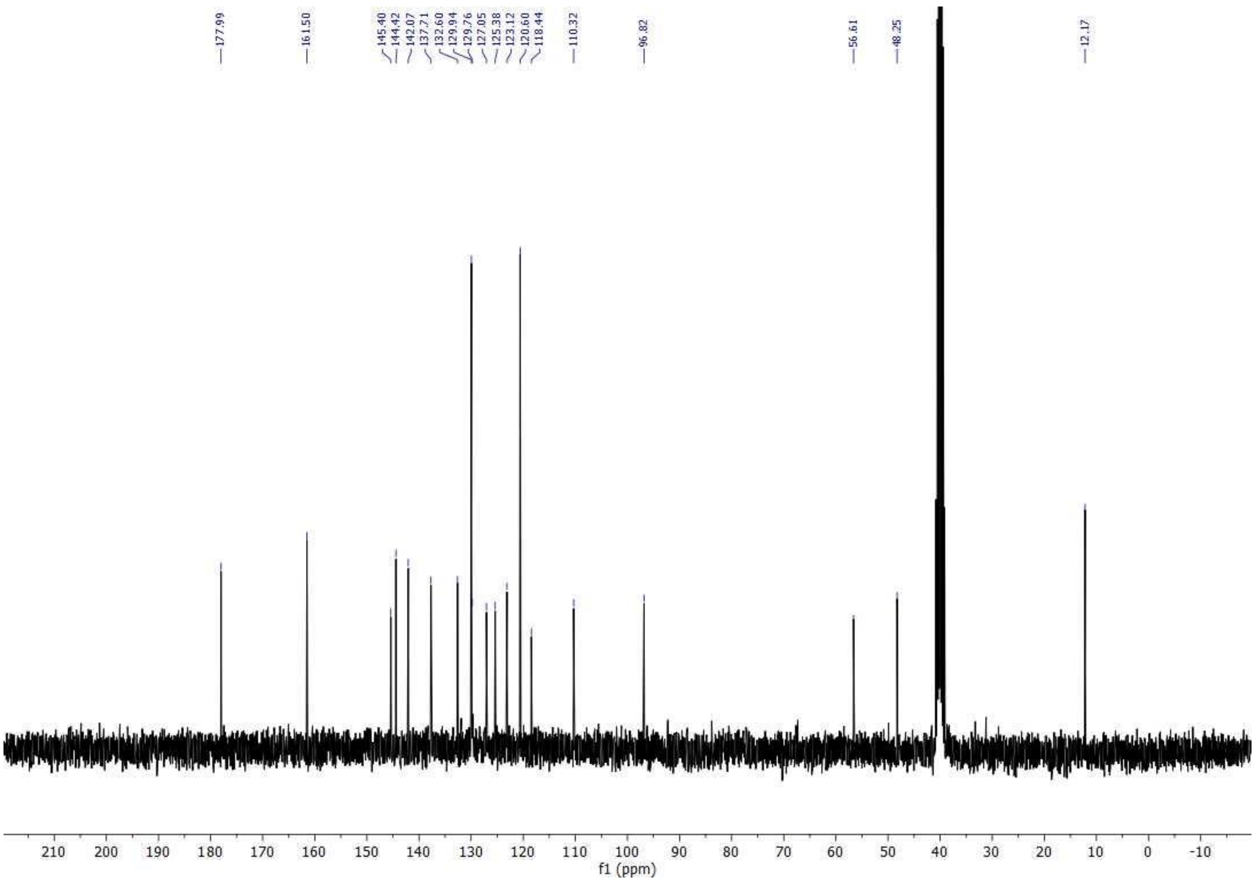

Supplement: RA-012-D1RA08182A-s001 [file RA-012-D1RA08182A-s001.pdf]
